# Comparative effects of β-glucan and mannan oligosaccharides on heat stress-induced inflammation: associations with gut barrier integrity and intestinal microbiota in mice

**DOI:** 10.3389/fnut.2026.1852838

**Published:** 2026-06-19

**Authors:** Ziye Zhang, Lei Feng, Bosen Zhang, Zilu Wang, Zhiyong Hu, Ruina Zhai

**Affiliations:** Key Laboratory of Efficient Utilization of Non-grain Feed Resources (Co-construction by Ministry and Province), Ministry of Agriculture and Rural Affairs, Shandong Provincial Key Laboratory of Animal Nutrition and Efficient Feeding, Department of Animal Science, Shandong Agricultural University, Taian, China

**Keywords:** gut barrier, heat stress, intestinal microbiota, mannan oligosaccharides, β-glucan

## Abstract

Heat stress poses serious threats to human and animal health by inducing systemic inflammation, oxidative stress, and intestinal barrier damage, yet the potential of functional food components in mitigating heat stress-associated health impairments remains insufficiently explored. This study used a chronic heat stress model in C57BL/6 J mice to compare the protective effects of β-glucan (BG) and mannan oligosaccharides (MOS) against heat stress-induced injury. The underlying mechanisms of each supplement were also systematically investigated. The results demonstrated that both BG and MOS effectively attenuated heat stress-induced body weight loss, elevated liver index, and systemic inflammatory responses, significantly reduced serum levels of interleukin-1β (IL-1β), interleukin-6 (IL-6), tumor necrosis factor-*α* (TNF-α), and heat shock protein 70 (HSP70), and restored antioxidant enzyme activity. Notably, BG exhibited superior efficacy in suppressing pro-inflammatory cytokines and restoring serum immunoglobulin A (IgA) levels. Regarding intestinal barrier integrity, both oligosaccharides markedly upregulated the colonic expression of tight junction proteins zonula occludens-1 (ZO-1), Claudin-1, and Occludin, decreased serum diamine oxidase (DAO) and lipopolysaccharide (LPS) levels, and partially alleviated heat stress-induced intestinal barrier disruption. Hepatic tissue analysis revealed that both BG and MOS ameliorated heat stress-induced hepatic inflammation and lipid metabolism dysfunction. This was achieved by suppressing TLR4 and iNOS expression while restoring the balance of CD36 and PPARα expression. Furthermore, fecal microbial diversity analysis revealed that MOS was associated with increased abundance of Lachnospiraceae-related taxa, which are known to include short-chain fatty acid-producing bacteria. These microbial changes may contribute to the maintenance of gut microecological homeostasis via distinct microbiota-associated pathways. Collectively, these findings suggest that BG and MOS may alleviate multi-level heat stress-induced damage, potentially in association with improved intestinal barrier-related markers, altered gut microbiota composition, and modulation of gut–liver axis-related responses, thereby providing preliminary evidence for their potential application as functional food components to address heat stress-related health challenges.

## 1 Introduction

Against the backdrop of global climate warming, the frequency and intensity of extreme heat events have been continuously rising, making the threat of heat stress to human and animal health increasingly impossible to ignore (1, 2). Heat stress refers to a series of physiological stress responses triggered by an imbalance in thermoregulatory capacity under high-temperature conditions, which can lead to elevated body temperature, reduced appetite, weight loss, and multi-organ dysfunction (3–5). Existing studies have demonstrated that heat stress can activate inflammatory signaling pathways, promoting the abundant secretion of pro-inflammatory cytokines such as IL-1β, IL-6, and TNF-*α*, while simultaneously inducing oxidative stress and suppressing humoral immune function, thereby causing multi-level damage to overall health (6–9).

The intestine is one of the core target organs of heat stress-induced injury (10, 11). Tight junction proteins, including ZO-1, Claudin-1, and Occludin, located between intestinal epithelial cells collectively constitute the physical intestinal barrier, maintaining effective separation between the intestinal contents and the body’s internal environment (12). Heat stress can directly damage intestinal epithelial cells and disrupt the expression and localization of tight junction proteins, leading to a significant increase in intestinal permeability (13, 14). This allows harmful substances such as bacterial LPS to enter the bloodstream via the gut-liver axis, subsequently activating the hepatic TLR4/NF-κB inflammatory signaling pathway and triggering hepatic inflammation and lipid metabolism dysfunction (9, 15). Meanwhile, heat stress can also significantly disturb the composition and structure of the gut microbiota, where the expansion of opportunistic pathogens and the reduction of beneficial bacteria compound one another, further exacerbating the vicious cycle of intestinal barrier damage and systemic inflammation (16, 17). Therefore, nutritional intervention strategies centered on maintaining intestinal barrier integrity and stabilizing microbiota structure represent a promising approach to counteracting heat stress-induced damage.

Functional food components have attracted increasing attention in recent years as potential nutritional intervention strategies against inflammation and stress, owing to their natural origin, favorable safety profiles, and multi-target biological activities (18–20). BG is a natural polysaccharide widely found in the cell walls of yeast and fungi as well as in cereals (21), and has been demonstrated to possess significant immunomodulatory, anti-inflammatory, and antioxidant activities (22–24). BG can exert cytoprotective effects at multiple levels by binding to pattern recognition receptors such as Dectin-1 and TLR2 on the surface of immune cells, thereby negatively regulating the NF-κB and MAPK inflammatory signaling pathways, as well as activating the Nrf2/HO-1 antioxidant pathway (25, 26). MOS are functional oligosaccharides extracted from yeast cell walls or plants such as konjac (27–29). As a classic prebiotic, MOS can improve gut microbiota composition and reinforce mucosal barrier function by competitively blocking the adhesion of pathogens to intestinal epithelial cells and selectively promoting the proliferation of short-chain fatty acid-producing bacteria (30–32). Although BG and MOS have been relatively well studied in the fields of intestinal health and immune regulation, their intervention effects on intestinal barrier damage, systemic inflammation, hepatic lipid metabolism dysfunction, and gut microbiota dysbiosis under heat stress conditions remain unclear, and systematic head-to-head comparative studies of the two components under equivalent heat stress models are scarce.

Although both BG and MOS have demonstrated protective effects on intestinal health individually, their mechanistic distinctions—particularly under the same pathological context—remain poorly understood. BG primarily appears to be associated with direct interactions with pattern recognition receptors such as Dectin-1 and TLR2 on immune cells, whereas MOS may predominantly act as a prebiotic substrate associated with selective modulation of gut microbiota composition. These fundamentally different action modes suggest that they may protect intestinal barrier integrity via non-overlapping pathways, yet no study has directly compared their differential efficacy and mechanisms under heat stress conditions. Therefore, the present study was designed not only to evaluate the protective effects of BG and MOS against heat stress-induced intestinal barrier dysfunction, systemic inflammation, hepatic injury, and gut microbiota dysbiosis, but also to systematically compare their differential regulatory mechanisms, thereby providing a mechanistic basis for their complementary or targeted application as functional food ingredients.

## 2 Materials and methods

### 2.1 Materials and reagents

Normal saline (IN9000, 0.9%, sterile), β-glucan (G9341, ≥ 80.0%), and mannan oligosaccharides (M9950, 93%) were purchased from Solarbio (Beijing, China). The BG product (purity ≥80.0%) was further characterized by the manufacturer (Solarbio, Beijing, China) as containing ≤10% protein and lipid impurities and ≤10% other yeast-derived polysaccharides (e.g., mannan fragments and chitin residues), with no other biologically active compounds reported. The MOS product had a purity of 93%. The potential biological impact of these minor impurities is discussed in Section 4. Enzyme-linked immunosorbent assay (ELISA) kits for IL-1β (catalog no. H009), IL-6 (catalog no. H007), interleukin-10 (IL-10, catalog no. H007), TNF-*α* (catalog no. H052), IgA (catalog no. H108), immunoglobulin G (IgG, catalog no. H106), HSP70 (catalog no. H264), malondialdehyde (MDA, catalog no. H003), glutathione peroxidase (GSH-Px, catalog no. H005), superoxide dismutase (SOD, catalog no. A001), DAO (catalog no. A088), LPS (catalog no. H255), and non-esterified fatty acids (NEFA, catalog no. A042) were obtained from the Nanjing Jiancheng Bioengineering Institute (Nanjing, China). Antibodies against plasmalemma vesicle-associated protein (PV1/PLVAP, catalog no. GB113141) and ZO-1 (catalog no. GB15195) were purchased from Servicebio (Wuhan, China). Primary antibodies for Western blotting were as follows: ZO-1 (#5406), Hsp70 (#4872), NLRP3 (#15101), and GAPDH (#2118) from Cell Signaling Technology (CST); *β*-Tubulin (ab6046), Bax (ab32503), and FGF21 (ab171941) from Abcam.

### 2.2 Experimental animals and design

All animal experimental protocols in this study were approved by the Laboratory Animal Welfare and Animal Experiment Ethics Review Committee of Shandong Agricultural University (approval no. SDAUA-2025-278). A total of 50 eight-week-old male C57BL/6 J mice were individually housed, with one mouse per cage, in temperature- and humidity-controlled rooms under a 12 h:12 h light–dark cycle with ad libitum access to food and water; the dietary composition and nutritional content of the mouse chow are provided in Supplementary Table 1. Following one week of acclimatization, mice were weighed and randomly assigned to four groups (*n* = 10 per group) using a weight-based stratified randomization strategy to ensure comparable baseline body weights: CON (maintained at 23 °C with 50% relative humidity), HS (exposed to 39 °C with 70% relative humidity for 2 h/day), HBG (exposed to 39 °C with 70% relative humidity for 2 h/day with BG supplementation), and HMOS (exposed to 39 °C with 70% relative humidity for 2 h/day with MOS supplementation). The CON and HS groups received 200 μL of normal saline daily by oral gavage, while the HBG and HMOS groups received 200 μL of BG solution (60 mg/mL) and MOS solution (20 mg/mL) daily by oral gavage, respectively. The doses of BG and MOS were selected based on previously published studies investigating polysaccharide interventions in mouse models, with endpoints related to intestinal barrier function and inflammatory responses (33, 34). The experiment lasted 14 days, during which food intake and body weight were recorded daily. At the end of the experiment, mice were anesthetized by continuous inhalation of isoflurane (3–5% for induction, 1.5–2% for maintenance, delivered in 100% oxygen). After confirming adequate anesthesia by the absence of pedal withdrawal reflex, blood samples were collected by retro-orbital blood collection into pro-coagulation tubes (serum separation tubes). The collected blood was allowed to clot at room temperature for 30 min, followed by centrifugation at 3,000 × g for 15 min at 4 °C. The resulting serum was carefully aspirated and stored at −80 °C until further analysis. Mice were subsequently euthanized by cervical dislocation, and death was confirmed by the cessation of heartbeat and respiration. Following euthanasia, the colon was excised and its length measured, and major organs were dissected and weighed to calculate organ indices. Adipose tissue was fixed in 4% paraformaldehyde for histological analysis, while the liver, colon, and other tissues were snap-frozen in liquid nitrogen and stored at −80 °C for subsequent analyses.

### 2.3 Determination of serum markers

Serum levels of IL-1β, IL-6, interleukin-10 (IL-10), TNF-*α*, IgA, IgG, HSP70, MDA, GSH-Px, SOD, DAO, LPS, and NEFA were determined according to the manufacturer’s instructions of the corresponding ELISA kits.

### 2.4 Quantitative real-time PCR (qRT-PCR) analysis of mouse samples

Total RNA was isolated from liver and colon tissue samples, and complementary DNA (cDNA) was synthesized using the Evo M-MLV RT Mix Kit with gDNA Clean for qPCR Ver.2 (Accurate Biotechnology Co., Ltd., China). The primer sequences used in this study are listed in Supplementary Table 2 and Supplementary Figure S1. Quantitative real-time PCR was performed using the SYBR Green Premix Pro Taq HS qPCR Kit (Accurate Biotechnology Co., Ltd., China). The PCR cycling conditions consisted of an initial denaturation step at 95 °C for 30 s, followed by 40 amplification cycles of 95 °C for 5 s and 60 °C for 30 s. Glyceraldehyde-3-phosphate dehydrogenase (GAPDH) was used as the reference housekeeping gene for normalization.

### 2.5 Histopathological analysis

Adipose tissue was fixed in 4% paraformaldehyde, followed by sequential dehydration and paraffin embedding, and subsequently sectioned using a rotary microtome (Leica, Germany). Tissue sections were deparaffinized prior to staining and then stained with hematoxylin and eosin (H&E) solution. Tissue morphology was observed and photographed using a light microscope (Nikon, Japan).

### 2.6 Immunofluorescence

Colon samples were embedded and sectioned, and the sections were incubated overnight at 4 °C with anti-ZO-1 and anti-PV1 primary antibodies. Cell nuclei were subsequently counterstained with 4′,6-diamidino-2-phenylindole (DAPI) for 10 min at room temperature. Detection of fluorescent signals was performed using a fluorescence microscope (Nikon Eclipse Ci-L, Nikon Corporation, Kyoto, Japan). Fluorescence intensity was quantified using Image-Pro Plus 6.0 software (Media Cybernetics, Inc., USA). For each animal, three non-overlapping fields were randomly captured from representative tissue sections at 100 × magnification. Mean fluorescence intensity per field was measured, and the average value per animal was used as the statistical unit (*n* = 6 animals per group). The same exposure settings and image acquisition parameters were applied uniformly across all groups.

### 2.7 Microbial diversity analysis

Total genomic DNA was extracted from microbial communities using a commercial extraction kit, and the quality of the extracted DNA was assessed by 1% agarose gel electrophoresis. DNA concentration and purity were determined using a NanoDrop 2000 spectrophotometer. PCR amplification was performed using the barcoded forward primer 338F (5′-ACTCCTACGGGAGGCAGCAG-3′) and reverse primer 806R (5′-GGACTACHVGGGTWTCTAAT-3′) targeting the V3–V4 hypervariable region of the 16S rRNA gene (35), and sequencing libraries were constructed from the purified PCR products using the NEXTFLEX Rapid DNA-Seq Kit. Sequencing was carried out on the Illumina Nextseq2000 platform (Majorbio Bio-Pharm Technology Co., Ltd., Shanghai, China). All data analysis and visualization were performed on the Majorbio Cloud Platform.^1^

### 2.8 Western blot analysis

Hepatic and colonic tissues were homogenized in RIPA lysis buffer supplemented with protease and phosphatase inhibitor cocktails, followed by sonication. Lysates were allowed to stand at 4 °C for 5 min and then centrifuged at 12,000 × g for 10 min. Supernatants were collected and protein concentrations were determined using a BCA protein assay kit. Equal amounts of protein were separated by SDS-PAGE and transferred onto PVDF membranes. Membranes were blocked with blocking buffer at 37 °C for 30 min, followed by overnight incubation at 4 °C with primary antibodies against FGF21, NLRP3, HSP70, and β-tubulin (liver), or Bax, ZO-1, HSP70, and GAPDH (colon). Following TBST washes, membranes were incubated with HRP-conjugated secondary antibodies for 1 h at room temperature. Protein bands were visualized using an ECL detection system and quantified with ImageJ software. Protein expression levels were normalized to the respective loading control (β-tubulin or GAPDH).

### 2.9 Statistical analysis

One-way analysis of variance (one-way ANOVA) was performed and figures were generated using GraphPad Prism 10.1.2 (GraphPad Software, San Diego, CA, USA) to analyze phenotypic characteristics, serum biomarker concentrations, enzyme activities, and gene expression data. Multiple group comparisons were conducted using Tukey’s multiple range test. Results are expressed as mean ± standard error of the mean (SEM). A value of *p* < 0.05 was considered statistically significant. Some figures were created with BioGDP.com (36). Data on food intake and body weight were analyzed using two-way repeated-measures ANOVA, with treatment group (CON, HS, HBG, and HMOS) as the between-subject factor and time (days 1–14) as the within-subject repeated-measures factor. The Geisser–Greenhouse correction was applied to adjust degrees of freedom for the time factor. Post-hoc multiple comparisons were performed using Tukey’s test. All statistical analyses were conducted in GraphPad Prism (version 10.1; GraphPad Software, San Diego, CA, USA). Differences were considered statistically significant at *p* < 0.05.

## 3 Results

### 3.1 Effects of BG and MOS on food intake, body weight, and organ indices in heat-stressed mice

**Figure 1:**
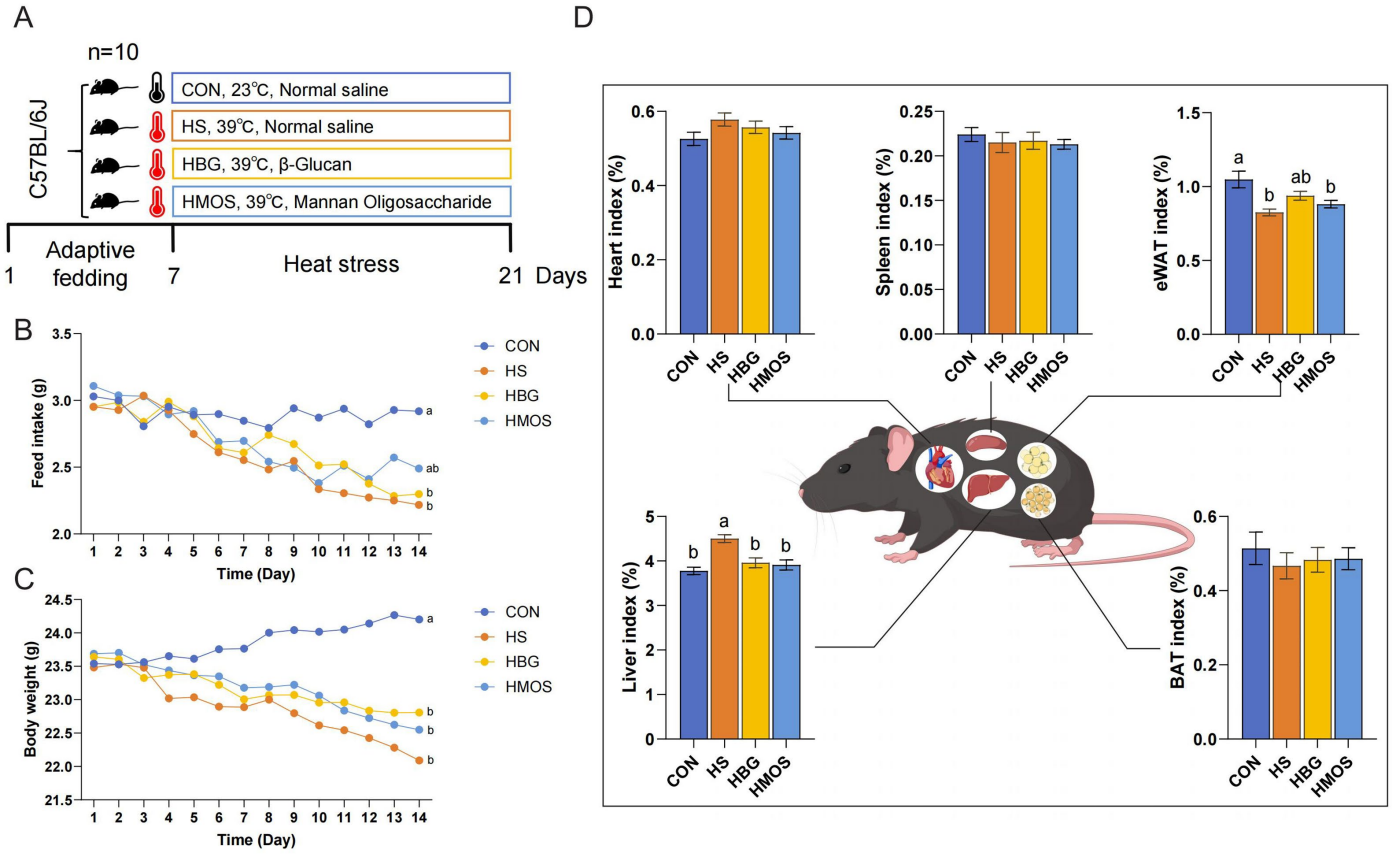
Effects of BG and MOS on food intake, body weight, and organ indices in heat-stressed mice. **(A)** Experimental design. **(B)** Changes in food intake over 14 days (*n* = 10). **(C)** Changes in body weight over 14 days (*n* = 10). **(D)** Cardiac, splenic, eWAT, BAT, and liver indices (*n* = 10). Data are expressed as mean ± SEM. Different letters indicate statistically significant differences (*p* < 0.05). The absence of a label indicates no significant difference (*p* > 0.05).

The experimental design is illustrated in Figure 1A. C57BL/6 J mice (*n* = 10/group) underwent a 7-day acclimatization period followed by 14 days of heat stress treatment, with daily oral gavage administration of normal saline (CON and HS groups), β-glucan (HBG group), or mannan oligosaccharides (HMOS group). Food intake in the CON group remained relatively stable throughout the experimental period, maintained at approximately 3.0 g/day. Following heat stress treatment, food intake in the HS group showed a continuous declining trend, with intake on day 14 being significantly lower than that of the CON group (*p* < 0.05). Food intake in the HBG and HMOS groups also exhibited a declining trend, although the magnitude of decline was slightly smaller than that observed in the HS group (Figure 1B). Body weight in the CON group increased continuously throughout the experiment, whereas the HS group showed a persistent decline in body weight, which was significantly lower than that of the CON group by day 14 (*p* < 0.05). The magnitude of body weight loss in both the HBG and HMOS groups was numerically smaller than that in the HS group (Figure 1C), suggesting that BG and MOS intervention partially attenuated heat stress-induced body weight loss. Regarding organ indices (Figure 1D), no significant differences were observed among groups in cardiac index, splenic index, or brown adipose tissue (BAT) index (*p* > 0.05). The liver index of the HS group was significantly higher than that of the CON, HBG, and HMOS groups (*p* < 0.05), while no significant differences were found among the CON, HBG, and HMOS groups, indicating that heat stress induced an increase in relative liver weight, which was effectively attenuated by BG and MOS intervention. The epididymal white adipose tissue (eWAT) index of the HS group was significantly lower than that of the CON group (*p* < 0.05), suggesting that heat stress led to a marked reduction in epididymal adipose tissue, while the restorative effects of BG and MOS intervention on eWAT appeared limited. It should be noted that the BG and MOS groups showed modestly higher feed intake than the HS group, which may have contributed to some of the observed protective effects.

### 3.2 Ameliorative effects of BG and MOS on systemic inflammation, oxidative stress, and immune dysfunction in heat-stressed mice

**Figure 2:**
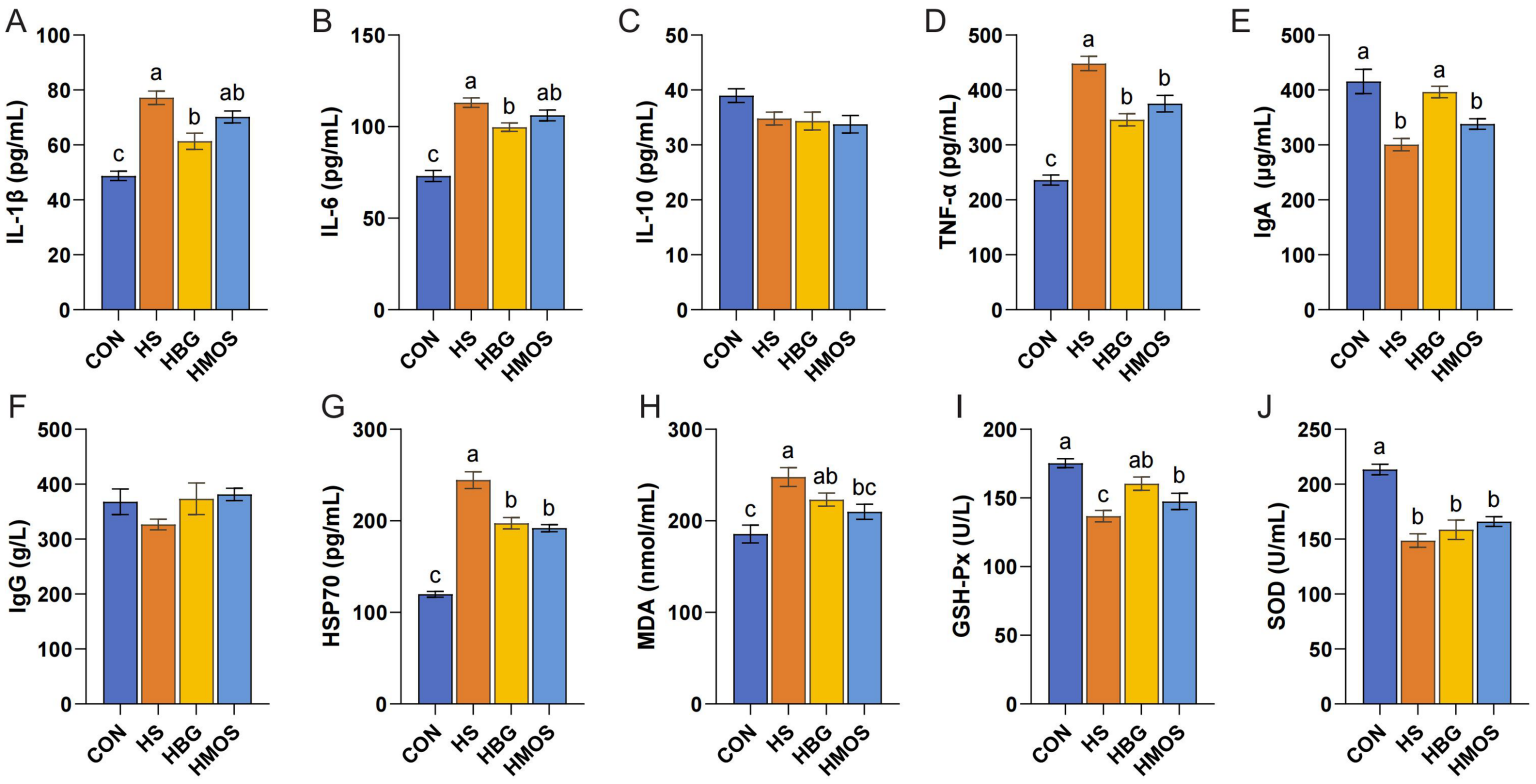
Effects of BG and MOS on serum inflammatory, immune, and antioxidant markers in heat-stressed mice. **(A–J)** Serum levels of IL-1β, IL-6, IL-10, TNF-α, IgA, IgG, HSP70, MDA, GSH-Px, and SOD (*n* = 6). Different letters indicate statistically significant differences (*p* < 0.05). The absence of a label indicates no significant difference (*p* > 0.05).

To further elucidate the effects of BG and MOS on inflammatory responses, oxidative stress, and immune function in heat-stressed mice, serum markers were measured across all groups. Regarding inflammatory indicators, compared with the CON group, serum levels of IL-1β, IL-6, and TNF-*α* were all significantly elevated in the HS group (*p* < 0.05; Figures 2A,B,D), indicating that heat stress successfully induced a pronounced systemic inflammatory response. Both BG and MOS intervention significantly reversed the heat stress-induced elevation in serum TNF-α (*p* < 0.05); furthermore, BG additionally reduced serum IL-1β and IL-6 levels significantly (*p* < 0.05), suggesting that BG is superior to MOS in suppressing heat stress-induced pro-inflammatory cytokines. No significant differences in serum IL-10 levels were observed among groups (*p* > 0.05; Figure 2C). With respect to serum immune indicators, serum IgA levels in the HS group were significantly lower than those in the CON group (*p* < 0.05; Figure 2E), while BG intervention effectively restored the heat stress-induced decline in IgA (*p* < 0.05), suggesting that heat stress impaired humoral immune function and that BG exerts a certain protective role in maintaining the mucosal immune barrier. No significant differences in serum IgG levels were detected among groups (*p* > 0.05; Figure 2F). Serum HSP70 levels, a hallmark protein of heat stress, were significantly higher in the HS group than in the CON group (*p* < 0.05). Both the HBG and HMOS groups showed significantly lower HSP70 levels compared with the HS group (*p* < 0.05), confirming successful establishment of the heat stress model and demonstrating that BG and MOS supplementation effectively alleviated heat stress-induced physiological responses in the animals. Regarding oxidative stress indicators (Figures 2H–J), heat stress significantly elevated serum MDA levels and markedly decreased GSH-Px and SOD activities (*p* < 0.05), indicating that heat stress induced substantial oxidative damage and disrupted the antioxidant defense system in mice. Both BG and MOS intervention significantly reduced serum MDA levels and elevated GSH-Px activity (*p* < 0.05), suggesting that both compounds can antagonize heat stress-induced oxidative damage by enhancing antioxidant enzyme activity and reducing lipid peroxidation.

Collectively, while both BG and MOS effectively reduced TNF-*α* and attenuated oxidative stress, BG demonstrated a broader anti-inflammatory profile by additionally suppressing IL-1β and IL-6 and restoring secretory IgA. This suggests that BG may exert a relatively more direct immunomodulatory effect associated with receptor-mediated signaling pathways, whereas MOS may primarily be associated with alleviation of systemic inflammation via microbiota-related regulatory mechanisms.

### 3.3 BG and MOS improved markers associated with intestinal barrier integrity

**Figure 3:**
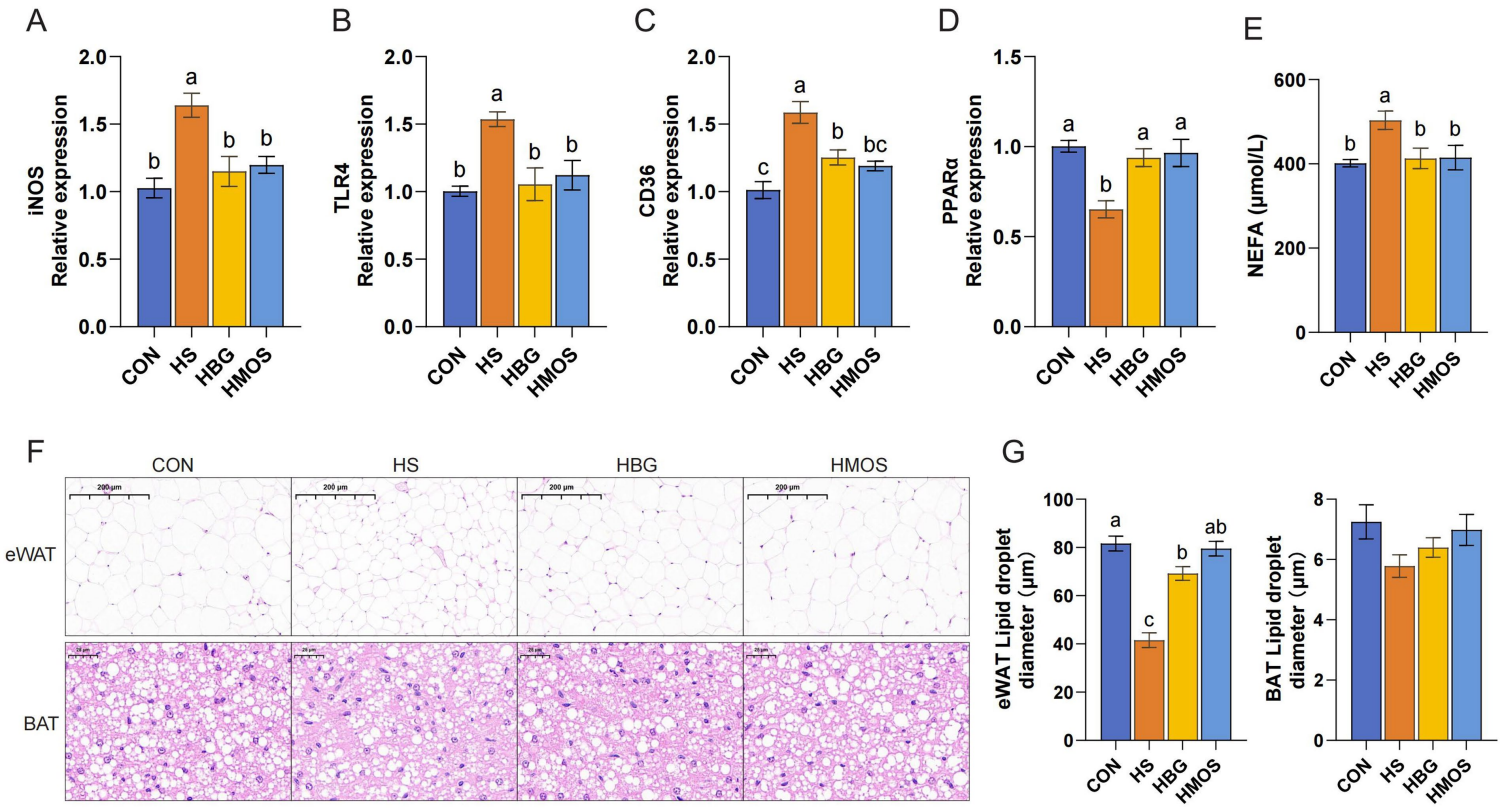
Effects of BG and MOS on colonic tissue in heat-stressed mice. **(A)** Representative images of mouse colonic tissue. **(B)** Statistical analysis of mouse colon length (*n* = 6). **(C)** Relative mRNA expression levels of IL-1β and IL-10 in mouse colonic tissue (*n* = 6). **(D)** Immunofluorescence images of ZO-1 and PV1 in mouse colonic tissue (100×). **(E)** Fluorescence intensity of ZO-1 and PV1 (*n* = 6). **(F)** Relative mRNA expression levels of ZO-1, Claudin-1, and Occludin in colonic tissue (*n* = 6). **(G)** Serum levels of DAO and LPS in mice (*n* = 6). Different letters indicate statistically significant differences (*p* < 0.05). The absence of a label indicates no significant difference (*p* > 0.05).

We evaluated the effects of BG and MOS on heat stress-induced colonic inflammation and barrier damage. Colon length was first measured, and the results showed (Figures 3A,B) that colon length in the HS group was significantly shorter than that in the CON group (*p* < 0.05), while colon length in both the HBG and HMOS groups was significantly greater than that in the HS group (*p* < 0.05), with no significant difference observed between the HBG group and the CON group. These findings indicate that heat stress induced colonic shortening, which was effectively attenuated by both BG and MOS intervention. With respect to the expression of colonic inflammation-related genes (Figure 3C), *IL-1β* mRNA levels in the HS group were significantly higher than those in the CON group, while *IL-10* mRNA levels were significantly lower than those in the CON group (*p* < 0.05), suggesting that heat stress induced a pronounced local inflammatory response in the colon. BG and MOS intervention restored the expression levels of *IL-1β* and *IL-10* to varying degrees, indicating that both compounds can effectively suppress heat stress-induced colonic inflammation.

Intestinal permeability assessment results (Figures 3D,E) showed that the CON group exhibited the highest colonic ZO-1 fluorescence signal intensity, while the HS group displayed a significantly attenuated ZO-1 fluorescence signal alongside a significantly enhanced PV1 fluorescence signal (*p* < 0.05), indicating that heat stress disrupted tight junction structures and increased intestinal permeability. ZO-1 fluorescence intensity in both the HBG and HMOS groups was significantly higher than that in the HS group, while PV1 fluorescence intensity was significantly lower than that in the HS group (*p* < 0.05), demonstrating that intervention with either oligosaccharide partially alleviated the damaged intestinal barrier. PCR analysis of tight junction-related genes in colonic tissue further corroborated these findings (Figure 3F), as mRNA expression levels of *ZO-1*, *Claudin-1*, and *Occludin* in the HS group were all significantly lower than those in the CON group (*p* < 0.05). Following BG and MOS intervention, *ZO-1* and *Occludin* expression levels were both significantly higher than those in the HS group (*p* < 0.05), with *Occludin* fully restored to CON group levels in both intervention groups, while *ZO-1* and *Claudin-1* showed partial recovery. These results suggest that both oligosaccharides can promote the transcriptional restoration of intestinal barrier-associated proteins.

To corroborate these transcriptional findings at the protein level, Western blot analysis was conducted to assess the expression of ZO-1, Bax, and HSP70 in colonic tissue (Supplementary Figures S2A,B). Consistent with the mRNA and immunofluorescence data, ZO-1 protein expression was significantly downregulated in the HS group compared with the CON group (*p* < 0.05), further confirming that heat stress impairs tight junction protein abundance at the translational level. Concurrently, the pro-apoptotic protein Bax was significantly upregulated in the HS group (*p* < 0.05), indicating that heat stress not only disrupted tight junction structure but also promoted colonic epithelial apoptosis, which may represent an additional mechanism underlying the observed barrier dysfunction. HSP70 protein expression was also markedly elevated in the HS group (*p* < 0.05), reflecting the degree of cellular heat stress sustained by colonic epithelial cells. Following BG and MOS supplementation, ZO-1 protein expression was significantly restored relative to the HS group (*p* < 0.05), while Bax and HSP70 protein levels were significantly reduced (*p* < 0.05), consistent with the gene expression results described above. These findings collectively demonstrate that BG and MOS reinforce intestinal barrier integrity not only by promoting the transcriptional recovery of tight junction proteins but also by attenuating heat stress-induced epithelial apoptosis at the protein level.

Serum DAO and LPS measurements were consistent with the aforementioned morphological and gene expression data (Figure 3G). Serum levels of both DAO and LPS in the HS group were significantly higher than those in the CON group (*p* < 0.05), indicating that heat stress markedly increased intestinal permeability and resulted in the translocation of bacterial endotoxins into the bloodstream. Both BG and MOS intervention significantly reduced serum DAO and LPS levels (*p* < 0.05); however, these values remained elevated compared with the CON group, suggesting that while both oligosaccharides effectively attenuated heat stress-induced increases in intestinal permeability, barrier function was not fully restored to baseline levels.

### 3.4 Effects of BG and MOS on hepatic inflammation, lipid metabolism, and adipose tissue morphology in heat-stressed mice

**Figure 4:**
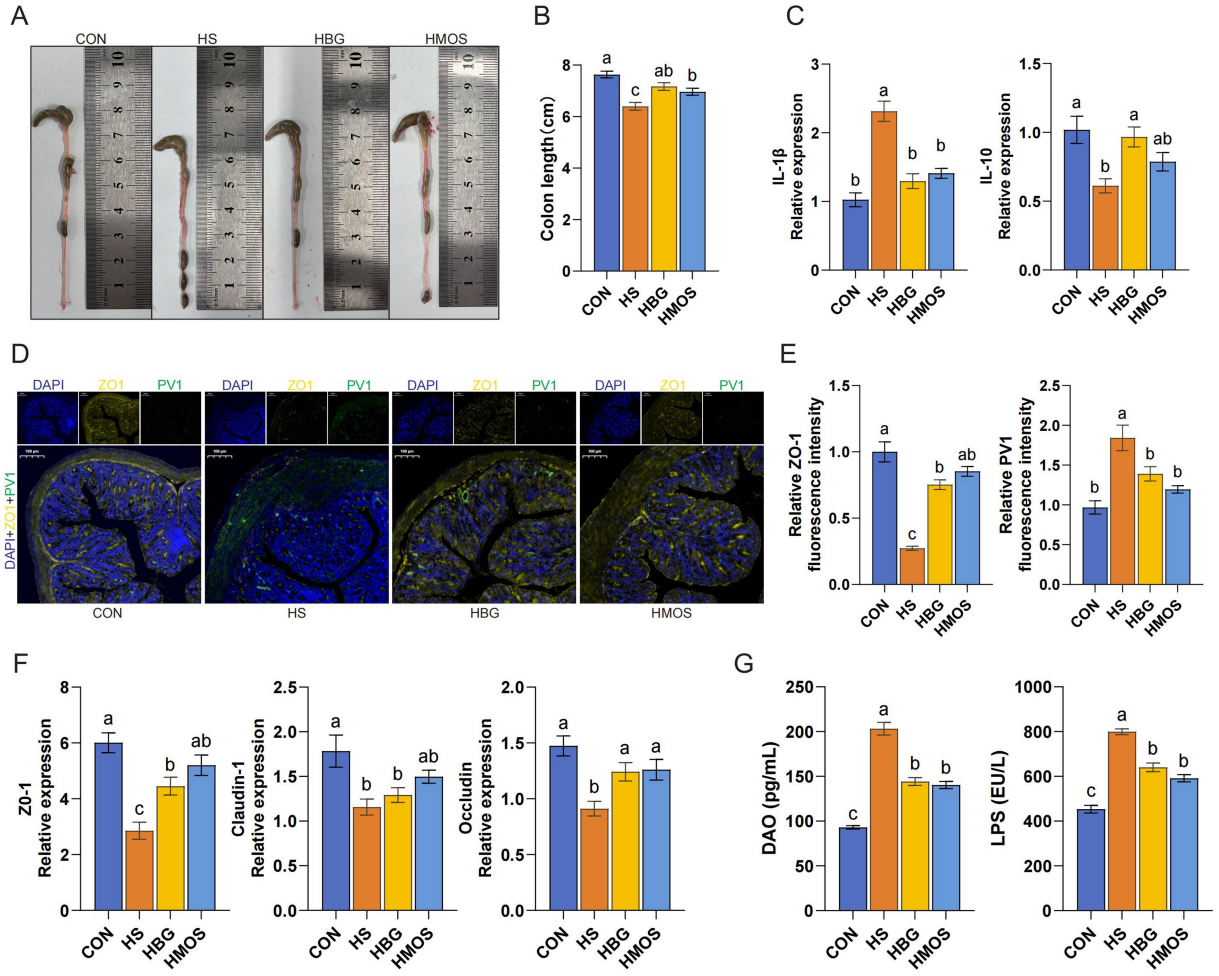
Effects of BG and MOS on hepatic and adipose tissues in heat-stressed mice. **(A)** Relative mRNA expression level of iNOS in hepatic tissue (*n* = 6). **(B)** Relative mRNA expression level of TLR4 in hepatic tissue (*n* = 6). **(C)** Relative mRNA expression level of CD36 in hepatic tissue (*n* = 6). **(D)** Relative mRNA expression level of PPARα in hepatic tissue (*n* = 6). **(E)** Serum NEFA levels in mice (*n* = 6). (F, G) Representative H&E staining images of eWAT and BAT (100 × and 400×) and lipid droplet diameter (*n* = 6 mice per group; values represent the mean of multiple lipid droplets measured per animal). Different letters indicate statistically significant differences (*p* < 0.05). The absence of a label indicates no significant difference (*p* > 0.05).

To further evaluate the intervention effects of BG and MOS on heat stress-induced metabolic dysregulation, the expression of inflammation- and lipid metabolism-related genes in hepatic tissue was examined. Regarding hepatic inflammatory indicators, mRNA expression levels of *iNOS* and *TLR4* in the HS group were both significantly higher than those in the CON group (*p* < 0.05; Figures 4A,B), while both genes were significantly downregulated in the HBG and HMOS groups compared with the HS group, with expression levels restored to levels comparable to those of the CON group. These findings indicate that heat stress induced a pronounced hepatic inflammatory response, which was effectively suppressed by both BG and MOS intervention.

To further validate these transcriptional findings at the protein level, Western blot analysis was performed to examine the expression of FGF21, NLRP3, and HSP70 in hepatic tissue (Supplementary Figures S2C,D). Consistent with the mRNA results, hepatic NLRP3 protein expression was significantly elevated in the HS group compared with the CON group (*p* < 0.05), corroborating the notion that heat stress activates the NLRP3 inflammasome and amplifies hepatic inflammatory signaling. FGF21 protein levels were likewise significantly upregulated in the HS group (*p* < 0.05), suggesting that heat stress concurrently triggers a hepatokine-mediated adaptive stress response. HSP70 protein expression was also markedly increased in the HS group (*p* < 0.05), reflecting the magnitude of cellular heat stress. Following HBG and HMOS supplementation, the protein expression of NLRP3, FGF21, and HSP70 was significantly reduced relative to the HS group (*p* < 0.05), consistent with the gene-level observations. Collectively, these results confirm that BG and MOS attenuate heat stress-induced hepatic inflammatory activation and cellular stress responses at both the transcriptional and translational levels.

With respect to hepatic lipid metabolism-related gene expression, *CD36* mRNA expression in the HS group was significantly higher than that in the CON group (*p* < 0.05; Figure 4C), and both BG and MOS intervention effectively suppressed the heat stress-induced overexpression of *CD36*. *PPARα* mRNA expression was significantly lower in the HS group compared with the CON, HBG, and HMOS groups (*p* < 0.05; Figure 4D), with no significant differences observed among the latter three groups, suggesting that heat stress suppressed the *PPARα*-mediated hepatic fatty acid *β*-oxidation pathway, while both BG and MOS intervention effectively restored *PPARα* expression. Serum NEFA measurements were consistent with the above gene expression data (Figure 4E); serum NEFA concentrations in the HS group were significantly higher than those in the CON, HBG, and HMOS groups (*p* < 0.05), with no significant differences among the latter three groups. This indicates that heat stress promoted peripheral lipolysis, leading to the release of large amounts of free fatty acids into the circulation, a process that was effectively attenuated by BG and MOS intervention. Taken together, the concurrent upregulation of CD36 and downregulation of PPARα suggest that heat stress may simultaneously promote hepatic fatty acid uptake while suppressing its oxidative utilization, thereby creating conditions for aberrant hepatic lipid metabolism disturbance. BG and MOS intervention appear to exert protective effects by bidirectionally correcting this metabolic imbalance.

We further performed H&E staining of epididymal white adipose tissue (eWAT) and brown adipose tissue (BAT) sections to assess lipid droplet changes. Lipid droplet diameter in eWAT of the HS group was significantly smaller than that in the CON group (*p* < 0.05; Figures 4F,G), while lipid droplet diameters in both the HBG and HMOS groups were significantly larger than those in the HS group (*p* < 0.05). These findings suggest that heat stress induced a marked reduction in visceral white adipose tissue lipid droplet size, and that BG and MOS intervention partially restored lipid droplet dimensions, which is consistent with the accelerated lipolysis reflected by the elevated serum NEFA levels. No significant differences in BAT lipid droplet diameter were observed among groups, indicating that the effects of heat stress on BAT morphology were limited.

### 3.5 Effects of BG and MOS on fecal microbial community structure in heat-stressed mice

**Figure 5:**
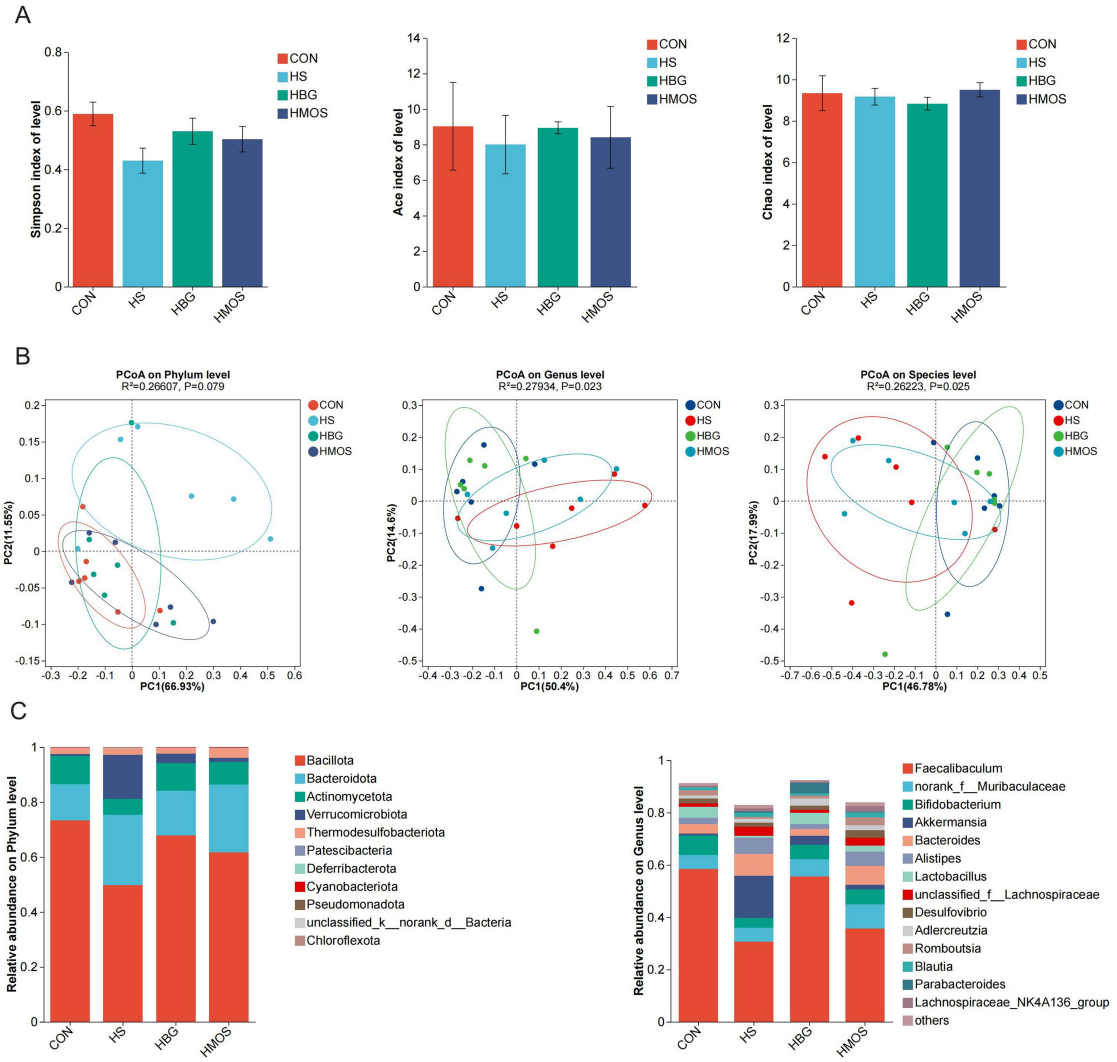
Effects of BG and MOS on fecal microbial diversity and species composition in heat-stressed mice. **(A)** Alpha diversity of fecal microbiota (*n* = 6). **(B)** Beta diversity of fecal microbiota (*n* = 6). Phylum level: pseudo-*F* = 2.42, R^2^ = 0.266, *p* = 0.059. Genus level: pseudo-*F* = 2.58, R^2^ = 0.279, *p* = 0.017. Species level: pseudo-*F* = 2.38, R^2^ = 0.262, *p* = 0.021. (PERMANOVA based on Bray-Curtis distances) **(C)** Species composition of fecal microbiota at the phylum and genus levels (*n* = 6).

To investigate the modulatory effects of BG and MOS on the gut microbial community in heat-stressed mice, 16S rRNA gene sequencing analysis was performed on fecal samples from all groups. Alpha diversity analysis (Figure 5A) revealed no significant differences in species diversity among groups (*p* > 0.05), suggesting that heat stress and oligosaccharide intervention had limited effects on the overall species richness of the gut microbiota.

Beta diversity principal coordinate analysis (PCoA) results (Figure 5B) showed that at the phylum level, samples from the CON and HS groups were completely separated, while samples from the HBG and HMOS groups shifted toward and overlapped with the CON group. At the genus and species levels, a relatively clear separation was also observed between the CON and HS groups, with the HBG group clustering more closely to and overlapping with the CON group.

Phylum-level compositional analysis (Figure 5C) revealed that the bacterial phyla with the highest relative abundance across all samples were Bacillota, Bacteroidota, Actinomycetota, and Verrucomicrobiota. At the genus level, *Faecalibaculum, norank_f_ Muribaculaceae*, *Bifidobacterium*, *Akkermansia*, and *Bacteroides* were the dominant genera across all groups.

**Figure 6:**
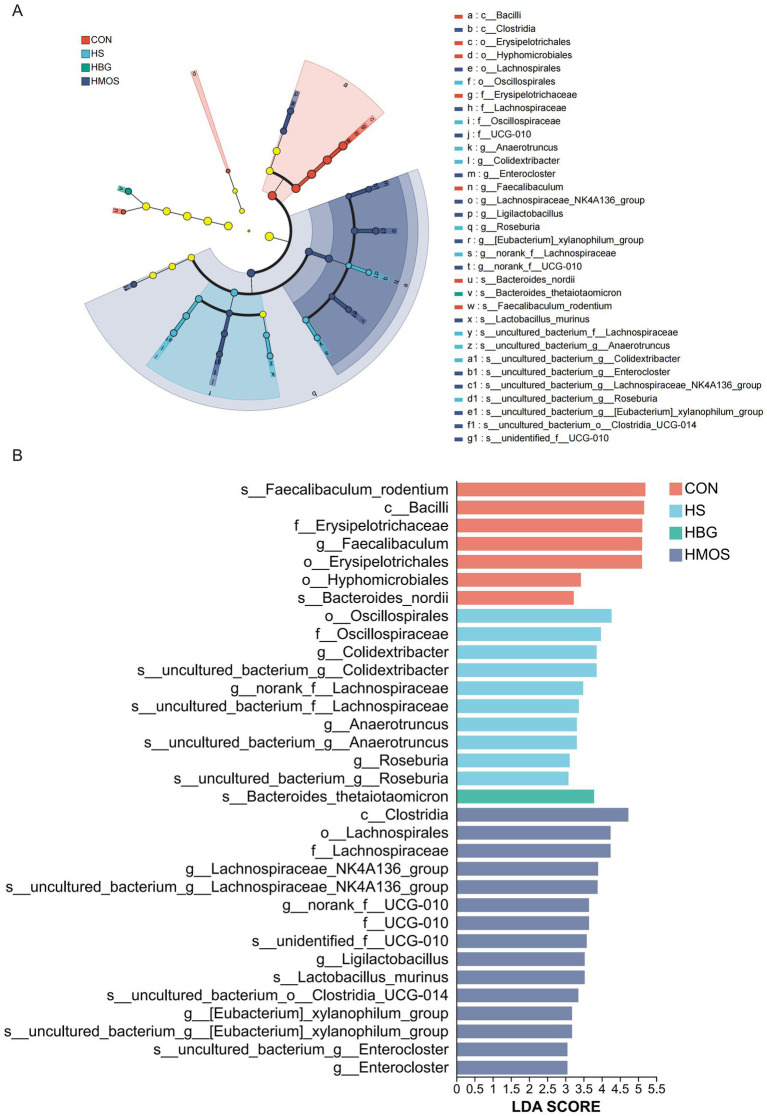
Effects of BG and MOS on the composition and structure of fecal microbial communities in heat-stressed mice. **(A)** LEfSe multilevel species cladogram (*p* < 0.05, LDA score > 3.0). **(B)** LDA discriminant scores.

To further characterize the differential features of microbial community composition among groups, LEfSe analysis (*p* < 0.05, LDA score > 3.0) identified a total of 33 discriminatory microbial taxa with inter-group distinguishing significance (Figures 6A,B). The characteristic microorganisms enriched in the CON group included *s_Faecalibaculum_rodentium*, *c_Bacilli*, *f_Erysipelotrichaceae*, *g_Faecalibaculum*, *o_Erysipelotrichales*, *o_Hyphomicrobiales*, and *s_Bacteroides_nordii*, most of which are common commensal bacteria in the intestines of healthy mice and are closely associated with the maintenance of intestinal immune homeostasis. Taxa significantly enriched in the HS group included *o_Oscillospirales*, *f_Oscillospiraceae*, *g_Colidextribacter*, *g_Anaerotruncus*, and *g_Roseburi*a, among others. The order *Oscillospirales*, family *Oscillospiraceae*, genus *Colidextribacter*, and genus Anaerotruncus have been largely associated with intestinal inflammation and barrier damage, and their increased abundance is consistent with the elevated intestinal permeability and exacerbated systemic inflammation observed in the HS group in this study. Only *s_Bacteroides_thetaiotaomicron* was significantly enriched in the HBG group. The HMOS group exhibited the richest profile of characteristic enriched taxa, including *c_Clostridia*, *o_Lachnospirales*, *f_Lachnospiraceae*, and *g_Lachnospiraceae_NK4A136_group*, among others. The substantial enrichment of short-chain fatty acid-producing bacteria centered on the family Lachnospiraceae and its related genera suggests that MOS intervention can selectively promote the proliferation of fermentative bacteria with intestinal protective functions, which mechanistically corroborates the intestinal barrier repair and colonic inflammation alleviation observed in the HMOS group.

BG preferentially enriched Bacteroides (predominantly assigned to *B. thetaiotaomicron* at the species level based on 16S V3-V4 amplicon sequencing, though species-level resolution requires further validation by shotgun metagenomics or qPCR), promoting mucus layer and Occludin restoration. MOS predominantly enriched Lachnospiraceae, a family widely associated with short-chain fatty acid production; however, as SCFAs were not directly quantified in the present study, whether this microbial shift translates into elevated butyrate and consequent barrier reinforcement via GPR109A-NF-κB signaling remains to be determined. Together, these results highlight potential mechanistic differences in microbial modulation between BG and MOS.

## 4 Discussion

A growing body of research has demonstrated that heat stress causes multifaceted damage to animal health by disrupting physiological homeostasis (37–39). In the present study, the continuous decline in food intake and body weight observed in heat-stressed mice is highly consistent with findings reported in previous studies (40, 41). Heat stress activates glutamatergic neurons in the parabrachial nucleus of the brainstem, transmitting thermosensory signals to hypothalamic tanycytes, which subsequently upregulate VEGFA expression and direct its release to the arcuate nucleus, thereby elevating the action potential threshold of orexigenic neurons and ultimately producing an anorexigenic effect (42). Furthermore, heat stress-induced systemic inflammation can further exacerbate feeding suppression through leptin resistance and impaired insulin signaling (43, 44). In the present study, the reductions in body weight and food intake in both the BG and MOS intervention groups were smaller than those in the HS group, suggesting that both compounds confer a degree of protection against heat stress-induced physiological impairment. This effect may be secondary to their ameliorative actions on intestinal health and systemic inflammation, rather than representing a direct action on the central appetite-regulating system (34, 45).

Systemic inflammation and oxidative stress are the core pathological features of heat stress-induced injury (46, 47). In the present study, heat stress significantly elevated serum levels of IL-1β, IL-6, TNF-α, and HSP70, reduced IgA levels, and induced MDA accumulation alongside decreased GSH-Px and SOD activities. This multidimensional evidence collectively reflects the pro-inflammatory state and comprehensive impairment of the antioxidant defense system induced by heat stress. The central mechanism by which heat stress activates systemic inflammation lies in the high-temperature-triggered activation of the NF-κB signaling pathway (48, 49), which drives macrophages and monocytes to secrete large quantities of pro-inflammatory cytokines, generating a cascading and amplified inflammatory response (50). Both BG and MOS intervention significantly reduced serum TNF-α levels, while BG additionally suppressed the elevation of IL-1β and IL-6, which is consistent with the previously reported mechanism by which β-glucan activates the MAPK pathway via Dectin-1 receptors and subsequently downregulates NF-κB inflammatory signaling (51, 52), although these specific signaling pathways were not directly examined in the present study. The overall superior anti-inflammatory efficacy of BG compared with MOS may be attributed to differences in their respective modes of pattern recognition receptor activation. The restoration of serum IgA was more pronounced in the BG group, which aligns with reports that β-glucan promotes immune responses in gut-associated lymphoid tissue and enhances secretory IgA production (53), suggesting that BG exerts more direct modulatory effects on mucosal immune function. Regarding oxidative stress, BG and MOS intervention significantly reduced MDA levels and restored GSH-Px activity, which may be related to their ability to activate the Nrf2/HO-1 antioxidant signaling pathway and upregulate the expression of endogenous antioxidant enzymes (54). As a hallmark protein of heat stress, the significant reduction in HSP70 observed in both the BG and MOS groups further indicates that these functional components can effectively alleviate heat stress-induced physiological responses, consistent with recent reports on the regulation of HSP70 expression by BG and MOS (55, 56).

Heat stress can impair the integrity of the intestinal epithelial barrier and significantly increase intestinal permeability, representing one of the key injury events in the pathological progression of heat stress (57, 58). In the present study, the damaging effects of heat stress on the intestinal barrier were confirmed across multiple dimensions, including colonic shortening, significant downregulation of mRNA expression of the tight junction proteins *ZO-1*, *Claudin-1*, and *Occludin*, enhanced PV1 fluorescence signal, and concurrent elevation of serum DAO and LPS levels, collectively pointing to dual damage of both the physical and functional intestinal barrier. The mechanisms by which heat stress disrupts the intestinal barrier operate at multiple levels: elevated temperatures can directly damage intestinal epithelial cells by activating the MLCK/MLC signaling pathway to induce cytoskeletal rearrangement, and through PKC-mediated phosphorylation disrupt the interaction between Occludin and ZO-1, thereby promoting the dissociation and degradation of tight junction proteins and ultimately leading to increased intestinal permeability (59–62). Heat stress also activates local intestinal inflammatory responses, whereby elevated colonic IL-1β can directly promote tight junction protein dissociation and increased intestinal permeability by activating the NF-κB/MLCK signaling pathway and inducing post-transcriptional degradation of *occludin* mRNA, while the concurrent decline in IL-10 weakens negative regulation of the aforementioned pro-inflammatory signals, further exacerbating barrier dysfunction (63, 64). BG and MOS intervention significantly restored tight junction protein expression and reduced serum DAO and LPS levels. Notably, Occludin was fully restored to CON group levels in both intervention groups, whereas ZO-1 and Claudin-1 showed only partial recovery, which may be related to differences in the functional positioning and regulatory mechanisms of each protein within the tight junction complex: Occludin is primarily regulated directly by phosphorylation signals, whereas ZO-1 is more sensitive to MLCK-mediated cytoskeletal signaling (65, 66). Although both oligosaccharides significantly reduced serum LPS levels, these values had not fully returned to control levels, suggesting that intestinal barrier function was only partially restored within the 14-day intervention period. Extending the intervention duration or employing combination therapies may further enhance the restorative effects, an issue that warrants deeper investigation in future studies.

The close connection between intestinal barrier damage and hepatic pathology is mediated through the portal venous system, with the gut-liver axis playing a central role in heat stress-induced liver injury (67, 68). In the present study, the elevation of serum LPS in the HS group was highly concordant with the upregulation of hepatic *TLR4* and *iNOS* expression, suggesting that gut-derived endotoxins entering the liver via the portal vein activated the TLR4/NF-κB signaling pathway and triggered hepatic inflammatory responses, consistent with the LPS-TLR4 signal transduction mechanism mediated by the gut-liver axis (69). Excessive activation of *iNOS* can inhibit mitochondrial respiratory chain function through the overproduction of nitric oxide, interfere with hepatocyte glucose metabolism and protein synthesis, mediate oxidative stress, and aggravate hepatocyte injury (70, 71). Concurrently, heat stress caused significant dysregulation at the level of hepatic lipid metabolism: upregulation of *CD36* expression indicates enhanced hepatic uptake capacity for peripheral free fatty acids (72, 73), while downregulation of *PPARα* expression reflects suppression of fatty acid *β*-oxidation (74), with both changes acting in concert to drive aberrant hepatic lipid metabolism disturbance. The significant elevation of serum NEFA levels further corroborates this inference from the perspective of peripheral lipolysis, and the reduction in epididymal white adipose tissue lipid droplet size is mutually corroborative with the NEFA elevation, indicating that heat stress accelerated the mobilization of visceral fat and delivered large quantities of free fatty acid substrates to the liver, resulting in the upregulation of *CD36* and downregulation of *PPARα* (75). By repairing the intestinal barrier and reducing LPS translocation into the bloodstream, BG and MOS intervention attenuated the continuous stimulation of hepatic TLR4 signaling by gut-derived endotoxins at the upstream level, while simultaneously restoring the expression balance of CD36 and PPARα, thereby conferring protection to the liver at both the inflammatory and lipid metabolic levels. The normalization of the liver index further confirms that both compounds exert substantive protective effects against heat stress-induced hepatic injury. No significant differences in BAT lipid droplet diameter were observed among groups, suggesting that the morphological impact of heat stress on brown adipose tissue thermogenic function was limited. As the primary function of BAT is to dissipate energy through UCP1-mediated non-shivering thermogenesis (76, 77), the metabolic regulatory effects of BG and MOS appear to be concentrated primarily on the white adipose mobilization and hepatic lipid metabolism axis.

The gut microbiota serves as an important regulator of intestinal barrier function and host immune metabolism (78), and alterations in its composition and structure are closely associated with heat stress-induced intestinal injury (79). Elucidating the microbiota-modulating effects of BG and MOS is therefore helpful for clarifying the potential mechanisms underlying their protective effects from a microbial perspective. Alpha diversity showed no significant differences among groups, and beta diversity at the phylum level was also not significant (*p* = 0.079). In contrast, beta diversity analysis at the genus and species levels revealed clear and significant differences between the heat stress and control groups, indicating that heat stress reshaped the microbial community at finer taxonomic resolutions. This is consistent with previously reported findings that gut microbiota composition changes under heat stress conditions without significant alterations in alpha diversity indices (80). LEfSe analysis further revealed the characteristics of heat stress-induced dysbiosis: taxa including *Oscillospirales*, *Colidextribacter*, and *Anaerotruncus* were significantly enriched in the HS group. Elevated abundance of *Colidextribacter* is closely associated with impaired intestinal permeability, as this genus has been shown to enhance cellular oxidative stress capacity and thereby participate in the pathological process of intestinal barrier damage (81). The enrichment of *Anaerotruncus* is also noteworthy, as its only known species, *Anaerotruncus colihominis*, has been reported to be associated with impaired barrier integrity and the occurrence of bacteremia under intestinal infection conditions (82). The elevated abundance of these taxa is mutually corroborative with the increased intestinal permeability and exacerbated systemic inflammation observed in the HS group of this study, suggesting that microbiota dysbiosis may be one of the important contributing factors to heat stress-induced intestinal barrier damage. The taxa enriched in the CON group, including *Faecalibaculum rodentium* and Bacilli-related taxa, are common commensal bacteria in the intestines of healthy mice. *Faecalibaculum rodentium* protects the intestine from inflammatory damage and maintains intestinal epithelial homeostasis by promoting histidine biosynthesis (83). BG and MOS interventions were associated with intestinal protective effects accompanied by enrichment of distinct functional microbial communities, with the two compounds exhibiting notably different microbiota-associated modulation patterns.

The BG group specifically enriched *Bacteroides thetaiotaomicron*, an important polysaccharide-degrading bacterium in the intestine (84) that can utilize dietary polysaccharides such as *β*-glucan as substrates, and whose enrichment reflects the selective promotional effect of BG on specific microbial populations. *Bacteroides thetaiotaomicron* can also promote the differentiation of colonic goblet cells and the expression of mucus-related genes, thereby reinforcing intestinal mucus barrier function (85). Furthermore, studies have shown that *Bacteroides thetaiotaomicron* can regulate host epithelial function by modulating the production of fucosylated glycan chains on intestinal epithelial cells, providing a potential microbiota-level explanation for the improvement in intestinal permeability observed with BG intervention (86).

The significant enrichment of Lachnospiraceae and the Lachnospiraceae NK4A136 group in the HMOS group is highly consistent with the prebiotic properties of mannan oligosaccharides. Lachnospiraceae represents an important short-chain fatty acid-producing bacterial community in the intestine, and a decline in its abundance is closely associated with pro-inflammatory states and intestinal barrier damage (87). Its fermentation product butyrate, as the primary energy source for colonic epithelial cells, can effectively attenuate intestinal inflammatory responses and repair intestinal barrier function by activating the GPR109A receptor to inhibit the NF-κB p65 signaling pathway (88). The enrichment of the Lachnospiraceae NK4A136 group has been demonstrated to be closely associated with improvements in intestinal barrier function (89). The HMOS group also showed concurrent enrichment of lactic acid bacteria including *Ligilactobacillus* and *Lactobacillus murinus*, which can inhibit pathogen colonization and maintain gut microecological stability through the production of organic acids, H₂O₂, and bacteriocins (90). BG and MOS were associated with intestinal protective effects accompanied by enrichment of distinct functional microbial communities, which may partly explain the differences observed between the two compounds in inflammation suppression and immune regulation in the present study.

Several limitations of this study should be acknowledged. The BG and MOS preparations differed in purity (≥80% vs. 93%), introducing a degree of uncertainty into direct comparisons. Residual components in the BG preparation were estimated at no more than 1.2 mg per mouse per day, a quantity unlikely to account for the observed protective effects at the administered dose. Furthermore, any functional contribution from mannan-containing residues would be expected to attenuate rather than accentuate the between-group differences. The distinct immunobiological profiles of BG and MOS are, moreover, consistent with their fundamental structural differences: BG is composed of glucose monomers linked via β-1,3 and β-1,6 glycosidic bonds and can adopt a triple-helical conformation recognized by pattern recognition receptors such as Dectin-1 and TLR2, thereby enabling direct modulation of innate immune signaling, whereas MOS consists of short-chain mannose oligomers connected through *α*-mannosidic linkages that render it a preferential fermentation substrate for butyrate-producing bacteria, supporting an indirect, microbiota-mediated mechanism of barrier protection (91, 92). These structural distinctions provide a mechanistic basis for the differential effects observed and further support the interpretation that the divergent outcomes reflect the intrinsic activities of the respective active components rather than preparation impurities. Nonetheless, future studies employing preparations of matched and higher purity, alongside dose–response designs calibrated to equimolar active ingredient quantities, would allow more rigorous attribution of effects to individual compounds (93, 94). The doses of BG and MOS were also not matched by mass, molarity, or fermentable carbohydrate content, and both interventions slightly altered voluntary feed intake relative to the heat-stressed controls. Consequently, observed differences in systemic inflammation, microbiota composition, and hepatic indices may partly reflect dose discrepancies or secondary effects of altered caloric intake. Future studies incorporating pair-fed control groups would help disentangle direct bioactive effects from those mediated by changes in energy consumption (95).

The absence of non-heat-stressed groups receiving BG or MOS supplementation alone (i.e., CON+BG and CON+MOS groups) precludes determination of whether either compound independently modulates gut microbiota, immune markers, or hepatic function under thermoneutral conditions, and such groups should be incorporated into future experimental designs. Additionally, although serum diamine oxidase activity, circulating lipopolysaccharide concentrations, ZO-1 immunofluorescence, and tight junction gene expression collectively suggest improved epithelial integrity, these parameters represent indirect indices of barrier function rather than direct functional measurements (78, 96). Direct assessments including FITC-dextran flux assays, Ussing chamber electrophysiology, or transepithelial electrical resistance measurements (97, 98) were not performed, and conclusions regarding barrier restoration should therefore be interpreted with caution.

Finally, the evidence for heat stress-induced hepatic lipid metabolic disturbance and its attenuation by BG or MOS rests primarily on alterations in CD36 and PPARα expression, serum non-esterified fatty acid concentrations, and liver index. Direct histological confirmation by hematoxylin–eosin or Oil Red O staining, hepatic triglyceride quantification, and assessment of hepatocellular injury via serum alanine aminotransferase and aspartate aminotransferase activities were not performed (99, 100). In the absence of these direct assessments, inferences regarding hepatic steatosis or lipid metabolic dysregulation remain tentative, and future investigations should incorporate comprehensive hepatic pathological and biochemical characterization to substantiate the observations reported here.

## 5 Conclusion

This study provides evidence that BG and MOS alleviate heat stress-induced damage at multiple levels. Taking intestinal barrier integrity as the primary point of intervention, both functional components restored tight junction protein expression and reduced gut-derived LPS translocation into the bloodstream, thereby severing the heat stress-induced TLR4/NF-κB inflammatory cascade at the upstream level and ameliorating hepatic inflammation and lipid metabolism dysfunction along the gut-liver axis. Notably, BG and MOS were associated with intestinal protective effects accompanied by enrichment of distinct microbial communities. BG was associated with increased abundance of Bacteroides-related taxa with potential barrier-supportive functions, whereas MOS was associated with enrichment of Lachnospiraceae-related taxa that are known to include short-chain fatty acid-producing bacteria. This differential microbiota-modulating pathway provides a microbiome-level explanation for the observed differences between the two compounds in anti-inflammatory and immunomodulatory efficacy. Whether the combined application of BG and MOS could achieve more comprehensive protective effects through complementary mechanisms warrants investigation in future studies. Furthermore, this study identifies a potential pattern of aberrant hepatic lipid metabolism disturbance characterized by concurrent CD36 upregulation and PPARα downregulation under sustained heat stress conditions, highlighting the underexplored role of heat stress as a contributing factor to altered hepatic lipid metabolic signaling. These findings provide a scientific basis for the application of BG and MOS as functional food components in addressing heat stress-related health challenges.

## Data Availability

The datasets presented in this study can be found in online repositories. The names of the repository/repositories and accession number(s) can be found at: https://www.ncbi.nlm.nih.gov/, PRJNA1438587.

## Footnotes

1
https://cloud.majorbio.com

## References

[1] Ebi KL Capon A Berry P Broderick C de Dear R Havenith G . Hot weather and heat extremes: health risks. Lancet. (2021) 398:698–708. doi: 10.1016/S0140-6736(21)01208-3 34419205

[2] Covert HH Abdoel Wahid F Wenzel SE Lichtveld MY. Climate change impacts on respiratory health: exposure, vulnerability, and risk. Physiol Rev. (2023) 103:2507–22. doi: 10.1152/physrev.00043.2022, 37326296

[3] Son J Kim HJ Hong EC Kang HK. Effects of stocking density on growth performance, antioxidant status, and meat quality of finisher broiler chickens under high temperature. Antioxidants. (2022) 11:871. doi: 10.3390/antiox11050871, 35624735 PMC9138006

[4] Chen L Thorup VM Kudahl AB Østergaard S. Effects of heat stress on feed intake, milk yield, milk composition, and feed efficiency in dairy cows: a meta-analysis. J Dairy Sci. (2024) 107:3207–18. doi: 10.3168/jds.2023-24059 38101736

[5] Bernal MA Schunter C Lehmann R Lightfoot DJ Allan BJM Veilleux HD . Species-specific molecular responses of wild coral reef fishes during a marine heatwave. Sci Adv. (2020) 6:eaay3423. doi: 10.1126/sciadv.aay3423, 32206711 PMC7080449

[6] Bagath M Krishnan G Devaraj C Rashamol VP Pragna P Lees AM . The impact of heat stress on the immune system in dairy cattle: a review. Res Vet Sci. (2019) 126:94–102. doi: 10.1016/j.rvsc.2019.08.011 31445399

[7] Calderwood SK Theriault JR Gong J. How is the immune response affected by hyperthermia and heat shock proteins? Int J Hyperth. (2005) 21:713–6. doi: 10.1080/02656730500340794, 16338853

[8] Song JH Kim KJ Chei S Seo YJ Lee K Lee BY. Korean red ginseng and Korean black ginseng extracts, JP5 and BG1, prevent hepatic oxidative stress and inflammation induced by environmental heat stress. J Ginseng Res. (2020) 44:267–73. doi: 10.1016/j.jgr.2018.12.005, 32148408 PMC7031738

[9] Deng ZC Yang JC Huang YX Zhao L Zheng J Xu QB . Translocation of gut microbes to epididymal white adipose tissue drives lipid metabolism disorder under heat stress. Sci China Life Sci. (2023) 66:2877–95. doi: 10.1007/s11427-022-2320-y 37480471

[10] Maseko T Dunshea FR Howell K Cho HJ Rivera LR Furness JB . Selenium-enriched Agaricus bisporus mushroom protects against increase in gut permeability ex vivo and up-regulates glutathione peroxidase 1 and 2 in hyperthermally-induced oxidative stress in rats. Nutrients. (2014) 6:2478–92. doi: 10.3390/nu6062478, 24962481 PMC4073163

[11] Liang H Liu N Wang R Zhang Y Chen J Dai Z . N-acetyl serotonin alleviates oxidative damage by activating nuclear factor erythroid 2-related factor 2 signaling in porcine enterocytes. Antioxidants. (2020) 9:303. doi: 10.3390/antiox9040303, 32272634 PMC7222184

[12] Feng R Ma LJ Wang M Liu C Yang R Su H . Oxidation of fish oil exacerbates alcoholic liver disease by enhancing intestinal dysbiosis in mice. Commun Biol. (2020) 3:481. doi: 10.1038/s42003-020-01213-8, 32879433 PMC7468239

[13] Gupta A Chauhan NR Chowdhury D Singh A Meena RC Chakrabarti A . Heat stress modulated gastrointestinal barrier dysfunction: role of tight junctions and heat shock proteins. Scand J Gastroenterol. (2017) 52:1315–9. doi: 10.1080/00365521.2017.1377285, 28906161

[14] Koch F Thom U Albrecht E Weikard R Nolte W Kuhla B . Heat stress directly impairs gut integrity and recruits distinct immune cell populations into the bovine intestine. Proc Natl Acad Sci USA. (2019) 116:10333–8. doi: 10.1073/pnas.1820130116, 31064871 PMC6535017

[15] Xiao C Kong L Pan X Zhu Q Song Z Everaert N. High temperature-induced oxidative stress affects systemic zinc homeostasis in broilers by regulating zinc transporters and Metallothionein in the liver and jejunum. Oxidative Med Cell Longev. (2022) 2022:1427335. doi: 10.1155/2022/1427335, 35387265 PMC8979716

[16] Li L Wang M Chen J Xu Z Wang S Xia X . Preventive effects of *Bacillus licheniformis* on heat stroke in rats by sustaining intestinal barrier function and modulating gut microbiota. Front Microbiol. (2021) 12:630841. doi: 10.3389/fmicb.2021.630841, 33889138 PMC8055866

[17] He J He Y Pan D Cao J Sun Y Zeng X. Associations of gut microbiota with heat stress-induced changes of growth, fat deposition, intestinal morphology, and antioxidant capacity in ducks. Front Microbiol. (2019) 10:903. doi: 10.3389/fmicb.2019.00903, 31105682 PMC6498187

[18] Olivares-González L Velasco S Campillo I Salom D González-García E Soriano Del Castillo JM . Nutraceutical supplementation ameliorates visual function, retinal degeneration, and redox status in rd10 mice. Antioxidants. (2021) 10:1033. doi: 10.3390/antiox10071033, 34206804 PMC8300708

[19] Zhang L Yin M Feng X Ibrahim SA Liu Y Huang W. Anti-inflammatory activity of four triterpenoids isolated from Poriae cutis. Foods. (2021) 10:3155. doi: 10.3390/foods10123155, 34945705 PMC8700795

[20] Linares DM O'Callaghan TF O'Connor PM Ross RP Stanton C. *Streptococcus thermophilus* APC151 strain is suitable for the manufacture of naturally GABA-enriched bioactive yogurt. Front Microbiol. (2016) 7:1876. doi: 10.3389/fmicb.2016.01876, 27920772 PMC5118970

[21] Martiniano SE Fernandes LA Alba EM Philippini RR Tabuchi SCT Kieliszek M . A new approach for the production of selenium-enriched and probiotic yeast biomass from agro-industrial by-products in a stirred-tank bioreactor. Meta. (2020) 10:508. doi: 10.3390/metabo10120508, 33322101 PMC7764536

[22] Luo Z Ma L Zhou T Huang Y Zhang L Du Z . Beta-Glucan alters gut microbiota and plasma metabolites in pre-weaning dairy calves. Meta. (2022) 12:687. doi: 10.3390/metabo12080687, 35893252 PMC9332571

[23] Liu Y Tang Q Feng J Liu J Tang C Yan M . Effects of molecular weight on intestinal anti-inflammatory activities of β-D-glucan from Ganoderma lucidum. Front Nutr. (2022) 9:1028727. doi: 10.3389/fnut.2022.1028727, 36245525 PMC9557179

[24] Wang X Ji Y Jin D Qi J Hou X Zhao W . Natural polysaccharide β-Glucan protects against doxorubicin-induced cardiotoxicity by suppressing oxidative stress. Nutrients. (2022) 14:906. doi: 10.3390/nu14040906, 35215555 PMC8878312

[25] Li X Luo H Ye Y Chen X Zou Y Duan J . β glucan, a dectin 1 ligand, promotes macrophage M1 polarization via NF κB/autophagy pathway. Int J Oncol. (2019) 54:271–82. doi: 10.3892/ijo.2018.4630, 30431070

[26] Yu C Chen H Du D Lv W Li S Li D . β-Glucan from *Saccharomyces cerevisiae* alleviates oxidative stress in LPS-stimulated RAW264.7 cells via Dectin-1/Nrf2/HO-1 signaling pathway. Cell Stress Chaperones. (2021) 26:629–37. doi: 10.1007/s12192-021-01205-5, 33880723 PMC8275741

[27] Luo Y Li J Zhou H Yu B He J Wu A . The nutritional significance of intestinal Fungi: alteration of dietary carbohydrate composition triggers colonic fungal community shifts in a pig model. Appl Environ Microbiol. (2021) 87:e00038–21. doi: 10.1128/AEM.00038-21, 33712429 PMC8117771

[28] Tanihiro R Sakano K Oba S Nakamura C Ohki K Hirota T . Effects of yeast Mannan which promotes beneficial Bacteroides on the intestinal environment and skin condition: a randomized, double-blind, placebo-controlled study. Nutrients. (2020) 12:3673. doi: 10.3390/nu12123673, 33260560 PMC7761098

[29] Zhu D Yan Q Li Y Liu J Liu H Jiang Z. Effect of Konjac Mannan oligosaccharides on glucose homeostasis via the improvement of insulin and leptin resistance in vitro and in vivo. Nutrients. (2019) 11:1705. doi: 10.3390/nu11081705, 31344867 PMC6723648

[30] Ma J Shah AM Shao Y Wang Z Zou H Kang K. Dietary supplementation of yeast cell wall improves the gastrointestinal development of weaned calves. Anim Nutr. (2020) 6:507–12. doi: 10.1016/j.aninu.2020.06.003, 33364467 PMC7750790

[31] Du Y Fan Y Li X Chen F. Novel anti-inflammatory properties of mannose oligosaccharides in the treatment of inflammatory bowel disease via LGALS3 modulation. NPJ Biofilms Microbiomes. (2025) 11:26. doi: 10.1038/s41522-025-00648-3, 39920168 PMC11806110

[32] Chen J Yin J Xie H Lu W Wang H Zhao J . Mannan-oligosaccharides promote gut microecological recovery after antibiotic disturbance. Food Funct. (2024) 15:3810–23. doi: 10.1039/d4fo00332b, 38511344

[33] Ma Y Zhu L Ke H Jiang S Zeng M. Oyster (*Crassostrea gigas*) polysaccharide ameliorates obesity in association with modulation of lipid metabolism and gut microbiota in high-fat diet fed mice. Int J Biol Macromol. (2022) 216:916–26. doi: 10.1016/j.ijbiomac.2022.07.100, 35868410

[34] Zhang T Cheng T Geng S Mao K Li X Gao J . Synbiotic combination between *Lactobacillus paracasei* VL8 and Mannan-oligosaccharide repairs the intestinal barrier in the dextran sulfate sodium-induced colitis model by regulating the intestinal stem cell niche. J Agric Food Chem. (2024) 72:2214–28. doi: 10.1021/acs.jafc.3c08473, 38237048

[35] Yu Y Lee C Kim J Hwang S. Group-specific primer and probe sets to detect methanogenic communities using quantitative real-time polymerase chain reaction. Biotechnol Bioeng. (2005) 89:670–9. doi: 10.1002/bit.20347, 15696537

[36] Jiang S Li H Zhang L Mu W Zhang Y Chen T . Generic diagramming platform (GDP): a comprehensive database of high-quality biomedical graphics. Nucleic Acids Res. (2025) 53:D1670–6. doi: 10.1093/nar/gkae973, 39470721 PMC11701665

[37] Jaswal P Rana D Chaudhary R Basu J Bansal N Gupta S . Heat waves and health crises: the unseen threat of heat stress on multiple organ systems. J Therm Biol. (2026) 136:104375. doi: 10.1016/j.jtherbio.2026.104375, 41691782

[38] Song M Li Y Zhou Y Yan J Zhou X Gao Q . Nicotinamide mononucleotide supplementation improves the quality of porcine oocytes under heat stress. J Anim Sci Biotechnol. (2022) 13:68. doi: 10.1186/s40104-022-00716-0, 35706001 PMC9202089

[39] Zhou C Yang S Ka W Gao P Li Y Long R . Association of gut Microbiota with Metabolism in rainbow trout under acute heat stress. Front Microbiol. (2022) 13:846336. doi: 10.3389/fmicb.2022.846336, 35432278 PMC9007319

[40] Yi W Cheng J Wei Q Pan R Song S He Y . Effect of temperature stress on gut-brain axis in mice: regulation of intestinal microbiome and central NLRP3 inflammasomes. Sci Total Environ. (2021) 772:144568. doi: 10.1016/j.scitotenv.2020.144568, 33770895

[41] Wu JJ Zheng X Wu C Ma W Wang Y Wang J . Melatonin alleviates high temperature exposure induced fetal growth restriction via the gut-placenta-fetus axis in pregnant mice. J Adv Res. (2025) 68:131–46. doi: 10.1016/j.jare.2024.02.014, 38382594 PMC11785557

[42] Benevento M Alpár A Gundacker A Afjehi L Balueva K Hevesi Z . A brainstem-hypothalamus neuronal circuit reduces feeding upon heat exposure. Nature. (2024) 628:826–34. doi: 10.1038/s41586-024-07232-3, 38538787 PMC11041654

[43] Ellett MD Rhoads RP Hanigan MD Corl BA Perez-Hernandez G Parsons CLM . Relationships between gastrointestinal permeability, heat stress, and milk production in lactating dairy cows. J Dairy Sci. (2024) 107:5190–203. doi: 10.3168/jds.2023-24043, 38428497

[44] Lakhoo DP Brink N Radebe L Craig MH Pham MD Haghighi MM . A systematic review and meta-analysis of heat exposure impacts on maternal, fetal and neonatal health. Nat Med. (2025) 31:684–94. doi: 10.1038/s41591-024-03395-8, 39500369 PMC11835737

[45] Nie Q Sun Y Hu W Chen C Lin Q Nie S. Glucomannan promotes *Bacteroides ovatus* to improve intestinal barrier function and ameliorate insulin resistance. iMeta. (2024) 3:e163. doi: 10.1002/imt2.163, 38868507 PMC10989147

[46] Cantet JM Yu Z Ríus AG. Heat stress-mediated activation of immune-inflammatory pathways. Antibiotics. (2021) 10:1285. doi: 10.3390/antibiotics10111285, 34827223 PMC8615052

[47] Cui Q Jia K Li F Zheng J Wang F. Post-translational modifications in heat stress-related diseases. Front Mol Biosci. (2025) 12:1666874. doi: 10.3389/fmolb.2025.1666874, 41059386 PMC12497517

[48] Wang Z Zhu J Zhang D Lv J Wu L Liu Z. The significant mechanism and treatments of cell death in heatstroke. Apoptosis. (2024) 29:967–80. doi: 10.1007/s10495-024-01979-w, 38886312

[49] Wang Y Ren J Ren T Li B Ran J Wang H . Photothermal reprogramming of synovial M1 macrophages reshapes the pro-inflammatory microenvironment to reverse temporomandibular joint osteoarthritis. J Nanobiotechnol. (2026) 24:258. doi: 10.1186/s12951-026-04258-9, 41796353 PMC13081524

[50] Wu J Zeng H Zhang C Chen H Mo P Huang S . Bruceine a ameliorates ulcerative colitis via macrophage polarization: targeting HSP90-mediated IL-17 signaling and NF-κB activation. Phytomedicine. (2025) 147:157200. doi: 10.1016/j.phymed.2025.157200, 40907407

[51] He W Li Z. Deciphering immunoregulatory mechanisms and structure-guided biosynthesis of β-glucans. Carbohydr Polym. (2025) 368:124254. doi: 10.1016/j.carbpol.2025.124254, 40947233

[52] Liu L Feng J Lian Z Geng J Wang W Wang J . Structural characterization of β-glucans from five edible fungi and structure-activity relationship on Dectin-1/toll like receptors activation. Food Chem. (2025) 493:145969. doi: 10.1016/j.foodchem.2025.145969, 40848343

[53] Mio K Otake N Nakashima S Matsuoka T Aoe S. Ingestion of high β-Glucan barley flour enhances the intestinal immune system of diet-induced obese mice by prebiotic effects. Nutrients. (2021) 13:907. doi: 10.3390/nu13030907, 33799564 PMC7999470

[54] Ye R Guo J Yang Z Wang Z Chen Y Huang J . Somatostatin and Mannooligosaccharide modified selenium nanoparticles with dual-targeting for ulcerative colitis treatment. ACS Nano. (2025) 19:14914–30. doi: 10.1021/acsnano.5c00355, 40214514

[55] Sayed Y Hassan M Salem HM Al-Amry K Eid GE. Prophylactic influences of prebiotics on gut microbiome and immune response of heat-stressed broiler chickens. Sci Rep. (2023) 13:13991. doi: 10.1038/s41598-023-40997-7, 37634024 PMC10460421

[56] Ezzat W Mahrose KM Rizk AM Ouda MMM Fathey IA Othman SI . Impact of β-glucan dietary supplementation on productive, reproductive performance and physiological response of laying hens under heat stress conditions. Poult Sci. (2024) 103:103183. doi: 10.1016/j.psj.2023.103183, 37931401 PMC10654246

[57] Lian P Braber S Garssen J Wichers HJ Folkerts G Fink-Gremmels J . Beyond heat stress: intestinal integrity disruption and mechanism-based intervention strategies. Nutrients. (2020) 12:734. doi: 10.3390/nu12030734, 32168808 PMC7146479

[58] Dokladny K Zuhl MN Moseley PL. Intestinal epithelial barrier function and tight junction proteins with heat and exercise. J Appl Physiol. (2016) 120:692–701. doi: 10.1152/japplphysiol.00536.2015, 26359485 PMC4868372

[59] Dokladny K Moseley PL Ma TY. Physiologically relevant increase in temperature causes an increase in intestinal epithelial tight junction permeability. Am J Physiol Gastrointest Liver Physiol. (2006) 290:G204–12. doi: 10.1152/ajpgi.00401.2005 16407590

[60] Lambert GP. Stress-induced gastrointestinal barrier dysfunction and its inflammatory effects. J Anim Sci. (2009) 87:E101–8. doi: 10.2527/jas.2008-1339, 18791134

[61] Yu Z Cantet JM Paz HA Kaufman JD Orellano MS Ipharraguerre IR . Heat stress-associated changes in the intestinal barrier, inflammatory signals, and microbiome communities in dairy calves. J Dairy Sci. (2024) 107:1175–96. doi: 10.3168/jds.2023-23873, 37730180

[62] Sun M Li Q Zou Z Liu J Gu Z Li L. The mechanisms behind heatstroke-induced intestinal damage. Cell Death Discov. (2024) 10:455. doi: 10.1038/s41420-024-02210-0, 39468029 PMC11519599

[63] Al-Sadi RM Ma TY. IL-1beta causes an increase in intestinal epithelial tight junction permeability. J Immunol. (2007) 178:4641–9. doi: 10.4049/jimmunol.178.7.4641, 17372023 PMC3724221

[64] Kaminsky LW Al-Sadi R Ma TY. IL-1β and the intestinal epithelial tight junction barrier. Front Immunol. (2021) 12:767456. doi: 10.3389/fimmu.2021.767456, 34759934 PMC8574155

[65] Bewley MC Tash BR Tian F Flanagan JM. A complex affair: attraction and repulsion make occludin and ZO-1 function! Tissue Barriers. (2013) 1:e23496. doi: 10.4161/tisb.23496, 24665376 PMC3879131

[66] Yu D Marchiando AM Weber CR Raleigh DR Wang Y Shen L . MLCK-dependent exchange and actin binding region-dependent anchoring of ZO-1 regulate tight junction barrier function. Proc Natl Acad Sci USA. (2010) 107:8237–41. doi: 10.1073/pnas.0908869107, 20404178 PMC2889526

[67] Albillos A de Gottardi A Rescigno M. The gut-liver axis in liver disease: pathophysiological basis for therapy. J Hepatol. (2020) 72:558–77. doi: 10.1016/j.jhep.2019.10.003, 31622696

[68] Wang J Wang X Zhuo E Chen B Chan S. Gut liver axis in liver disease: from basic science to clinical treatment (review). Mol Med Rep. (2025) 31:10. doi: 10.3892/mmr.2024.13375, 39450549 PMC11541166

[69] Carotti S Guarino MP Vespasiani-Gentilucci U Morini S. Starring role of toll-like receptor-4 activation in the gut-liver axis. World J Gastrointest Pathophysiol. (2015) 6:99–109. doi: 10.4291/wjgp.v6.i4.99, 26600967 PMC4644892

[70] Wang YY Chen MT Hong HM Wang Y Li Q Liu H . Role of reduced nitric oxide in liver cell apoptosis inhibition during liver damage. Arch Med Res. (2018) 49:219–25. doi: 10.1016/j.arcmed.2018.09.001, 30269965

[71] Liu Q Rehman H Krishnasamy Y Ramshesh VK Theruvath TP Chavin KD . Role of inducible nitric oxide synthase in mitochondrial depolarization and graft injury after transplantation of fatty livers. Free Radic Biol Med. (2012) 53:250–9. doi: 10.1016/j.freeradbiomed.2012.05.012, 22609250 PMC3392495

[72] Nauli AM Nassir F Zheng S Yang Q Lo CM Vonlehmden SB . CD36 is important for chylomicron formation and secretion and may mediate cholesterol uptake in the proximal intestine. Gastroenterology. (2006) 131:1197–207. doi: 10.1053/j.gastro.2006.08.012, 17030189 PMC1994908

[73] Koonen DP Jacobs RL Febbraio M Young ME Soltys CL Ong H . Increased hepatic CD36 expression contributes to dyslipidemia associated with diet-induced obesity. Diabetes. (2007) 56:2863–71. doi: 10.2337/db07-0907, 17728375

[74] Régnier M Polizzi A Smati S Lukowicz C Fougerat A Lippi Y . Hepatocyte-specific deletion of Pparα promotes NAFLD in the context of obesity. Sci Rep. (2020) 10:6489. doi: 10.1038/s41598-020-63579-3, 32300166 PMC7162950

[75] Zhang S Williams KJ Verlande-Ferrero A Chan AP Su GB Kershaw EE . Acute activation of adipocyte lipolysis reveals dynamic lipid remodeling of the hepatic lipidome. J Lipid Res. (2024) 65:100434. doi: 10.1016/j.jlr.2023.100434, 37640283 PMC10839691

[76] Cannon B Nedergaard J. Brown adipose tissue: function and physiological significance. Physiol Rev. (2004) 84:277–359. doi: 10.1152/physrev.00015.2003, 14715917

[77] Wang Q Liu Y Xu Y Jin Y Wu J Ren Z. Comparative transcriptome and Lipidome analyses suggest a lipid droplet-specific response to heat exposure of brown adipose tissue in normal and obese mice. Life Sci. (2022) 299:120540. doi: 10.1016/j.lfs.2022.120540, 35398332

[78] Bischoff SC Barbara G Buurman W Ockhuizen T Schulzke JD Serino M . Intestinal permeability--a new target for disease prevention and therapy. BMC Gastroenterol. (2014) 14:189. doi: 10.1186/s12876-014-0189-7, 25407511 PMC4253991

[79] Tang Z Yang Y Wu Z Ji Y. Heat stress-induced intestinal barrier impairment: current insights into the aspects of oxidative stress and endoplasmic reticulum stress. J Agric Food Chem. (2023) 71:5438–49. doi: 10.1021/acs.jafc.3c00798, 37012901

[80] Wen C Li S Wang J Zhu Y Zong X Wang Y . Heat stress alters the intestinal microbiota and Metabolomic profiles in mice. Front Microbiol. (2021) 12:706772. doi: 10.3389/fmicb.2021.706772, 34512584 PMC8430895

[81] Di Vincenzo F Del Gaudio A Petito V Lopetuso LR Scaldaferri F. Gut microbiota, intestinal permeability, and systemic inflammation: a narrative review. Intern Emerg Med. (2024) 19:275–93. doi: 10.1007/s11739-023-03374-w, 37505311 PMC10954893

[82] Lightfoot YL Yang T Sahay B Zadeh M Cheng SX Wang GP . Colonic immune suppression, barrier dysfunction, and dysbiosis by gastrointestinal *bacillus anthracis* infection. PLoS One. (2014) 9:e100532. doi: 10.1371/journal.pone.0100532, 24945934 PMC4063899

[83] Zeng W Wu J Xie H Xu H Liang D He Q . Enteral nutrition promotes the remission of colitis by gut bacteria-mediated histidine biosynthesis. EBioMedicine. (2024) 100:104959. doi: 10.1016/j.ebiom.2023.104959, 38215690 PMC10827402

[84] Wong JPH Chillier N Fischer-Stettler M Zeeman SC Battin TJ Persat A. *Bacteroides thetaiotaomicron* metabolic activity decreases with polysaccharide molecular weight. MBio. (2024) 15:e0259923. doi: 10.1128/mbio.02599-23, 38376161 PMC10936149

[85] Wrzosek L Miquel S Noordine ML Bouet S Joncquel Chevalier-Curt M Robert V . *Bacteroides thetaiotaomicron* and *Faecalibacterium prausnitzii* influence the production of mucus glycans and the development of goblet cells in the colonic epithelium of a gnotobiotic model rodent. BMC Biol. (2013) 11:61. doi: 10.1186/1741-7007-11-61, 23692866 PMC3673873

[86] Hooper LV Wong MH Thelin A Hansson L Falk PG Gordon JI. Molecular analysis of commensal host-microbial relationships in the intestine. Science. (2001) 291:881–4. doi: 10.1126/science.291.5505.881, 11157169

[87] Lobionda S Sittipo P Kwon HY Lee YK. The role of gut microbiota in intestinal inflammation with respect to diet and extrinsic stressors. Microorganisms. (2019) 7:271. doi: 10.3390/microorganisms7080271, 31430948 PMC6722800

[88] Chen G Ran X Li B Li Y He D Huang B . Sodium butyrate inhibits inflammation and maintains epithelium barrier integrity in a TNBS-induced inflammatory bowel disease mice model. EBioMedicine. (2018) 30:317–25. doi: 10.1016/j.ebiom.2018.03.030, 29627390 PMC5952406

[89] Stadlbauer V Engertsberger L Komarova I Feldbacher N Leber B Pichler G . Dysbiosis, gut barrier dysfunction and inflammation in dementia: a pilot study. BMC Geriatr. (2020) 20:248. doi: 10.1186/s12877-020-01644-2, 32690030 PMC7372911

[90] Xie H Yu S Tang M Xun Y Shen Q Wu G. Gut microbiota dysbiosis in inflammatory bowel disease: interaction with intestinal barriers and microbiota-targeted treatment options. Front Cell Infect Microbiol. (2025) 15:1608025. doi: 10.3389/fcimb.2025.1608025, 40654576 PMC12245916

[91] Brown GD Gordon S. Immune recognition. A new receptor for beta-glucans. Nature. (2001) 413:36–7. doi: 10.1038/35092620, 11544516

[92] Ramberg JE Nelson ED Sinnott RA. Immunomodulatory dietary polysaccharides: a systematic review of the literature. Nutr J. (2010) 9:54. doi: 10.1186/1475-2891-9-54, 21087484 PMC2998446

[93] Zhu F Du B Xu B. A critical review on production and industrial applications of beta-glucans. Food Hydrocoll. (2016) 52:275–88. doi: 10.1016/j.foodhyd.2015.07.003

[94] Vetvicka V Vetvickova J. Comparison of immunological effects of commercially available β-glucans. Appl Sci Rep. (2014) 1:1–7. doi: 10.7243/2054-9903-1-2, 36720374

[95] Hall KD Guo J. Obesity energetics: body weight regulation and the effects of diet composition. Gastroenterology. (2017) 152:e3:1718–27. doi: 10.1053/j.gastro.2017.01.052, 28193517 PMC5568065

[96] Turner JR. Intestinal mucosal barrier function in health and disease. Nat Rev Immunol. (2009) 9:799–809. doi: 10.1038/nri2653, 19855405

[97] Srinivasan B Kolli AR Esch MB Abaci HE Shuler ML Hickman JJ. TEER measurement techniques for in vitro barrier model systems. J Lab Autom. (2015) 20:107–26. doi: 10.1177/2211068214561025, 25586998 PMC4652793

[98] Clarke LL. A guide to Ussing chamber studies of mouse intestine. Am J Physiol Gastrointest Liver Physiol. (2009) 296:G1151–66. doi: 10.1152/ajpgi.90649.2008, 19342508 PMC2697950

[99] Kleiner DE Brunt EM Van Natta M Behling C Contos MJ Cummings OW . Nonalcoholic Steatohepatitis clinical research network. Design and validation of a histological scoring system for nonalcoholic fatty liver disease. Hepatology. (2005) 41:1313–21. doi: 10.1002/hep.20701, 15915461

[100] Giannini EG Testa R Savarino V. Liver enzyme alteration: a guide for clinicians. CMAJ. (2005) 172:367–79. doi: 10.1503/cmaj.1040752, 15684121 PMC545762

