# Supplementary material for: Comparative effects of β-glucan and mannan oligosaccharides on heat stress-induced inflammation: associations with gut barrier integrity and intestinal microbiota in mice
Source: Front Nutr. 2026 Jun 19;13:1852838. doi: 10.3389/fnut.2026.1852838 (PMC13328099; doi:10.3389/fnut.2026.1852838)
Supplement: Supplementary file 1 [file Supplementary_file_1.pdf]

Supplementary Material

1 Supplementary Tables

Table. S1 Ingredient composition and nutrient levels of the basal diet

Ingredient composition, g	g	kcal
Casein, 30 Mesh	140	560
L-Cystine	1.8	7.2
Corn Starch	495.692	1983
Maltodextrin 10	125	500
Sucrose	100	400
Cellulose	50	0
Soybean Oil	40	360
t-Butylhydroquinone	0.008	0
Mineral Mix S10022M	35	0
Vitamin Mix V10037	10	40
Choline Bitartrate	2.5	0
Total	1000	3850.2

**nutrient level, %**	**g/100g**	**kcal%**
Protein	14.2	14.7
Carbohydrate	73.1	75.9
Fat	4	9.4
Total		100
kcal/gm	3.85	

Table S2. The primers used in the mouse experiment

Gene	Sequence of forward primers (5' to 3'）	Sequence of reverse primers (5' to 3')
IL-1β	GCCACCTTTTGACAGTGATGA	AAGGTCCACGGGAAAGACAC
IL-10	CAGTGGAGCAGGTGAAGAGT	CGGAGAGAGGTACAAACGAGG
ZO-1	GACGCTTCCCGGACTTTTGT	GTCACTGTGTGCTGTTCCCA
Occludin	CTTTCCTTAGGCGACAGCGG	GAGTACGCTGGCTGAGAGAG
Claudin-1	AGACCTGGATTTGCATCTTGGTG	TGCAACATAGGCAGGACAAGAGTTA
iNOS	CAGCACAGGAAATGTTTCAGC	TAGCCAGCGTACCGGATGA
TLR4	AGTTTCAGCTACCAAGCCT	GTTCTAGTTGCTCTAAGCCCAT
CD36	ATGGGCTGTGATCGGAACTG	TTTGCCACGTCATCTGGGTTT
PPARα	ACCACTACGGAGTTCACGCATG	GAATCTTGCAGCTCCGATCACAC
GAPDH	TGTGTCCGTCGTGGATCTGA	TTGCTGTTGAAGTCGCAGGAG

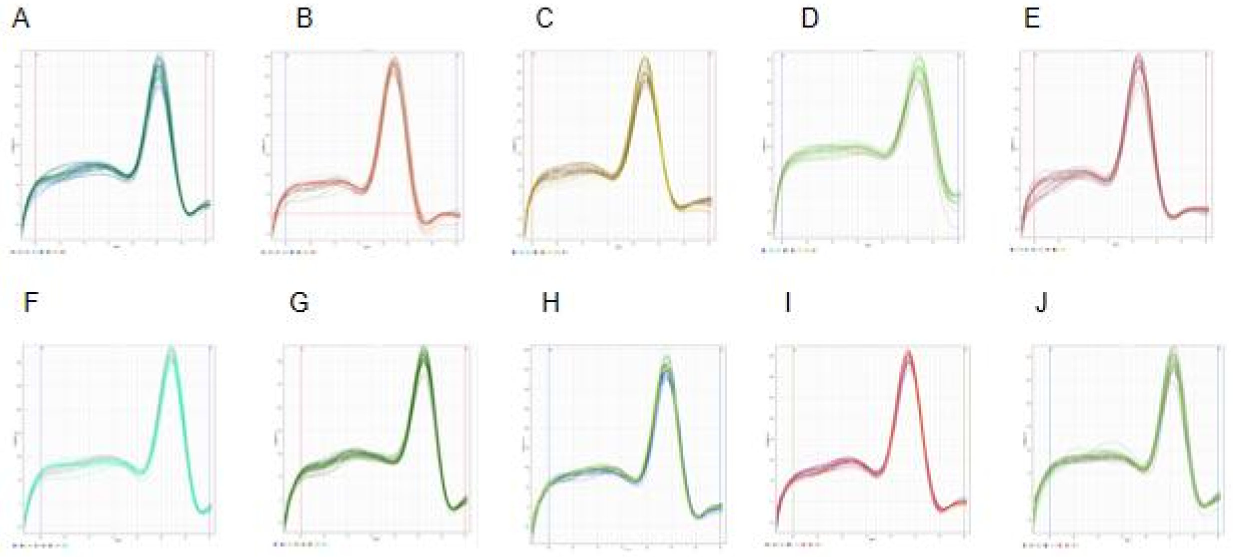

Figure S1. Melt curves of qPCR amplification for target genes.(A-J) IL-1β, IL-10, ZO-1, Occludin, Claudin-1, iNOS, TLR4, CD3, PPARα, GAPDH.

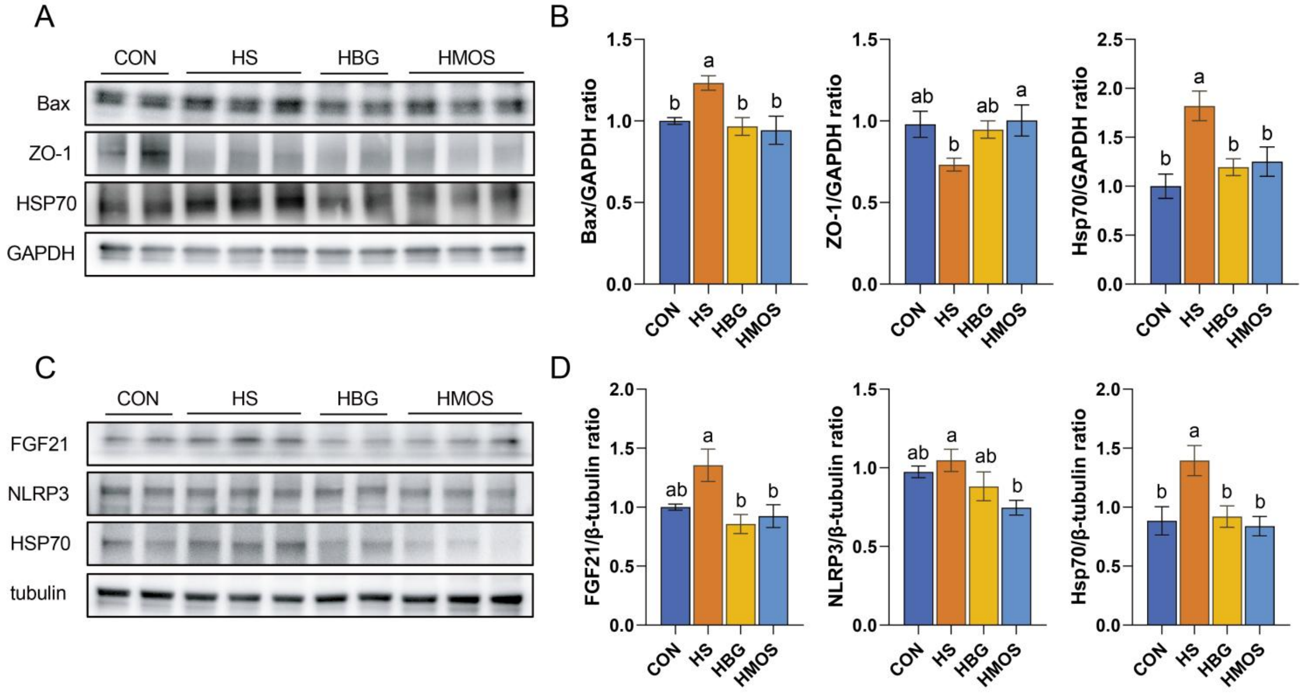

Figure S2. Western blot analysis of liver and intestinal proteins. (A–B) Representative western blot bands and quantification of Bax, ZO-1, and HSP70 protein expression in mouse colon tissue (n = 3). (C–D) Representative western blot bands and quantification of FGF21, NLRP3, and HSP70

protein expression in mouse liver tissue (n = 3). Data are presented as mean ± SEM. Different letters indicate statistically significant differences ($P < 0.05$), while the absence of letters indicates no significant difference ($P > 0.05$).
